# Measuring socioeconomic gaps in nutrition and early child development in Bolivia

**DOI:** 10.1186/s12939-020-01197-1

**Published:** 2020-07-20

**Authors:** Pablo Celhay, Sebastian Martinez, Cecilia Vidal

**Affiliations:** 1 School of Government, Pontificia Universidad Católica de Chile and Millennium Nuclei for the Study of the Life Course and Vulnerability, Avda. Vicuña Mackenna 4860 – Macul, Santiago, Chile; 2 Inter-American Development Bank, 1300 New York Avenue, NW, Washington, DC 20577 USA

**Keywords:** Child nutrition, Early childhood development, Socioeconomic gap, Bolivia

## Abstract

**Background:**

A large body of evidence shows that socioeconomic status (SES) is strongly associated to children’s early development, health and nutrition. Few studies have looked at within sample differences across multiple measures of child nutrition and development. This paper examines SES gaps in child nutritional status and development in Bolivia using a representative sample of children 0–59 months old and a rich set of outcomes, including micronutrient deficiencies, anthropometic measures, and gross motor and communicative development.

**Methods:**

We construct direct and *proxy* measures of living standards based on household expenditures and on ownership of assets combined with access to services and dwelling characteristics. The data for this study come from a nationally representative household survey in Bolivia that contains information on health, nutrition, and child development tests. We used a regression framework to assess the adjusted associations between child development outcomes and socioeconomic status, after controlling for other demographic factors that might affect child’s development. The SES gap in child development was estimated by OLS. To explore when the development gaps between children in different socioeconomic groups start and how they change for children at different ages, we analyze the differences in outcomes between the poorest (Q1) and richest (Q5) quintiles by child’s age by estimating kernel weighted local polynomial regressions of standardized scores for all child development indicators.

**Results:**

There are large and statistically significant differences in all anthropometrics z-scores between children in Q5 and children in Q1: height for age (0.95 SD), weight for age (0.70 SD), and weight for height (0.21 SD). When we divide the sample into children at the bottom and top consumption quintiles the results show that 68.6% of children in the poorest quintile are anemic. While this percentage falls to 40.9% for children in the richest quintile, it remains high compared to other countries in the region. The prevalence of vitamin A deficiency is 29.9% for children in the richest quintile and almost 10 percentage points higher for those at the bottom quintile (39.0%); the prevalence of Iron deficiency for children in the top and bottom quintiles is 16.4% and 23.8%, respectively. Compared to the most deprived quintile, children in the wealthiest quintile are less likely to have iron deficiency, anemia, to be stunted, and to have a risk of delays in gross motor and communicative development. At age three, most of these gaps have increased substantially. Our findings are robust to the choice of socioeconomic measurement and highlight the need for targeted policies to reduce developmental gaps.

**Conclusion:**

These findings highlight the need for targeted public policies that invest in multiple dimensions of child development as early as possible, including health, nutrition and cognitive and verbal stimulation. From a policy perspective, the large socioeconomic gaps in nutrition outcomes documented here reinforce the need to strengthen efforts that tackle the multiple causes of malnutrition for the poorest.

## Introduction

In 2010, an estimated 249.4 million children worldwide risked failing to reach their developmental potential because of stunting and poverty [1]^1^. Studies analyzing socioeconomic gradients in child health and early development show that poorer children are less likely to be healthy and well-nourished, achieve optimal cognitive abilities and adequately communicate with others [3–6]. These early disparities are carried through life and have implications for educational attainment, income generation, adult health, risky behaviors^2^ and other dimensions of individual and social wellbeing [7–9]. Losses from poor cognitive and educational performance in poor children account for subsequent lower employability and earnings [10], high fertility and poorer parenting practices for their children in the future [2].

There are several mediating factors related to poverty that prevent children’s optimal development^3^. Black et al. [12] find that undernutrition and unstimulating household environments contribute to deficits in children’s health and development that affect adulthood economic outcomes. Micronutrient deficiencies such as iron and vitamin A have also been found to affect cognitive, motor, and socioemotional development [11, 13]. Vitamin A deficiency in children is responsible for over one million child deaths annually, and is the leading cause of preventable child blindness in developing countries [14]. Likewise, Iron deficiency has been associated with poorer child cognitive, motor, and social-emotional functions [11, 13]. Other social determinants of child development include access to clean water and sanitation, parents education, and access to quality early childhood development services [7].

In this paper, we explore socioeconomic gradients in nutrition and final child development outcomes in Bolivia using a large nationally representative sample of children under five years of age. Although we analyze gaps for all outcomes individually, we also test the association between SES and final child development after accounting for nutritional status as a mediating factor. Various elements make Bolivia an interesting case study. While the country has experienced important improvements in child nutrition and poverty indicators in the last decade, disparities between socioeconomic groups remain high. In 2016, one of every ten children living in low poverty municipalities was stunted, while this rate almost tripled for high poverty municipalities. At the same time, Bolivia has the second highest prevalence rate of anemia in the Latin American region, with 67% of children under five in the poorest municipalities presenting some level of anemia [15]^4^. Also, Bolivia’s three ecological regions (highlands, valleys and lowlands) differ in multiple environmental and socio-cultural factors, providing an opportunity to explore whether these regional differences might contribute or modify SES gaps in child development.

Our study contributes to the growing literature of inequalities in child development in several aspects. The richness of our data allows us to look at child development outcomes, including gross motor and communication development, as well as nutrition risk factors, such as undernutrition, anemia and micronutrient deficiencies, within the same representative sample of children. Another contribution of this paper is the use of novel data collected from dried blood samples to measure vitamin A and iron deficiencies. In addition, we measure SES through direct and *proxy* measures of living standards based on household expenditures and on ownership of assets combined with dwelling characteristics. The availability of detailed information to obtain multiple measures of SES is unusual for health surveys and in our study, it permits testing the robustness of our results. Thus, our study complements the existing body of literature that focuses on particular subpopulations of disadvantaged children [4] or analyzes SES gaps in child development across countries using a single SES indicator and measure of child development [5].

We additionally explore the heterogeneity of our results by looking at changes in SES gaps across age groups and ecological regions. These analyses are particularly relevant as they provide information for the design and targeting of evidence-informed early child development interventions that can benefit the poorest and most disadvantaged children in Bolivia.

The remainder of the paper is organized as follows. Section 2 describes the data, explains the construction of SES and child nutrition and development outcomes, and describes the strategy to measure SES gradients. Section 3 presents SES gaps by developmental domain and explores how SES gaps differ across ages. Section 4 presents robustness checks of our results to the choice of the socioeconomic indicator. Section 5 concludes.

## Methods

### Data sources

The data for this study come from the Health and Nutrition Evaluation Survey (ESNUT). The ESNUT is a one-round large household survey jointly implemented between April and December 2012 by the Ministry of Health and the Ministry of Development Planning^5^. Its sample consists of a multistage cluster design that provides representative statistics for households with children under five years old at the national and regional levels, and allows also urban-rural disaggregation within regions^6^.

The ESNUT collected data using three main questionnaires: i) a household questionnaire used to collect basic sociodemographic characteristics of household members (family composition, education, labor participation and income), physical housing quality, access to basic services, household asset ownership, and household expenditure; ii) a woman’s questionnaire, administered to all women aged 14 to 49 years, used to collect birth histories for the five year period preceding the survey (2007-2012) with detailed information on prenatal, birth and postnatal care; and iii) a child’s questionnaire used to gather data from all children under five, including health status, measures of height and weight, hemoglobin levels, immunizations, nutritional practices, and history of visits to health providers (reported and recorded in health cards). To assess developmental progress in children younger than 3 years, the ESNUT included the gross motor and communicative modules of a child development screening questionnaire. In a random subsample of children between 6 and 23 months, the survey additionally collected blood samples to measure vitamin A and iron levels in blood [16].

The full sample of analysis contains 11,358 children from 0 to 59 months in 8,433 households (2,456 urban and 5,977 rural). The subsamples of analysis have 5,763 children from 0 to 36 months with available gross motor and communicative information, and 1,610 children between 6 and 23 months with information for the analysis of vitamin A and iron deficiencies.

### Assessment of nutritional status and early child development

To measure children’s nutritional status, the survey included anthropometric indicators and hemoglobin level to identify the presence of anemia, and blood concentration of vitamin A and iron to identify micronutrient deficiencies. Child growth indicators and standardized scores (z-scores) were constructed following the World Health Organization (WHO) guidelines [17] for the three most common anthropometric indices: height for age, weight for age, and weight for height. The z-score system expresses the anthropometric value as the number of standard deviations from the median of the WHO reference population^7^^,^^8^. In addition, we computed indicators of prevalence of chronic malnutrition (stunting), underweight, and overweight, based on height for age and weight for age standard cutoff values of below or above two standard deviations from the reference median.

To measure anemia the ESNUT obtained levels of hemoglobin in blood for each child between 3 and 59 months using a *HemoCue®* test for the photometric detection of hemoglobin. This method has been used extensively in household surveys in developing countries, including the Demographic and Health Surveys. Presence of mild, moderate or severe anemia was then determined based on altitude-adjusted hemoglobin levels and standard cutoff values^9^.

One of ESNUT’s specific objectives was to assess deficits of key micronutrients in small children; specifically, vitamin A deficiency (VAD) and iron deficiency (ID). To measure VAD and ID, blood samples were obtained from a subsample of 2,000 children ages 6 to 23 months using the Dried Blood Spots (DBS) method. This method collects a few blood drops from a heel or finger prick that are then impregnated in filter paper and let to dry^10^. All DBS were rehydrated and analyzed in a laboratory using the Enzyme-Linked Immunosorbent Assay method (ELISA)^11^ [18]. The indicators to estimate concentration of Vitamin A and iron were the Retinol Binding Protein (RBP) and the Free Transferrin Receptor (sTfR), respectively. VAD was defined as RBP below 0.7μmol/l [19] and ID as sTfR above 8.3 mg/l. Excluding samples with low quality (damaged or small blood spots) and with indication of inflammation, the total sample size for micronutrient analysis was 1,655^12^.

Early child development was assessed through measures of gross motor and communicative development. The ESNUT used 11 age-specific survey questionnaires for children between 3.5 and 36.5 months^13^ which were based on the second edition of the Ages and Stages Questionnaires® (ASQ-2)^14^. The questionnaires contained items about tasks that the child is (or is not) able to perform according to his or her age. Most items were reported by the child’s caregiver, while some specific items were based on direct observation of the child. To increase scores’ variability, the survey added items of decreasing and increasing difficulty. Similar adaptations have been used in other studies [20–22]. The questionnaires’ language was adapted to the local context of Bolivia.

Each item had a score of 10, 5 or 0 depending on whether the child can perform the task always, sometimes, or never, respectively. Raw scores were constructed for each domain as the sum of scores across items. Because the population on which the ASQ was standardized (US children) was not considered an appropriate reference population for our sample, we constructed within sample or internally standardized scores adjusted by age. Following standard procedures, internal *z*-scores were constructed within the eleven age groups to have a mean of 0 and a SD of 1 (by subtracting the age-group specific mean of the raw score and dividing by the age-group specific SD).

### Measures of socioeconomic status

There are several ways to measure socioeconomic status from household survey information, including direct monetary measures (income or expenditure), and *proxy* measures such as composite indices derived from ownership of household assets and living conditions. Following existing literature on health inequalities [23], we used the rich information collected by ESNUT on household expenditures and asset ownership to construct alternative measures of SES and check the sensitivity of our results to the choice of the SES indicator.

The question of whether the choice of SES measure matters in the analysis of socioeconomic inequalities has been explored in the literature, yet without a conclusive answer. While some studies find that the choice between consumption and the asset index makes little difference to the measured degree of inequality [24], others argue that results are actually sensitive to the choice of SES measure [25], or even to the choice of assets and characteristics that are included in the wealth index [26]. Consistent with previous findings, the relationship between our consumption and wealth index is relatively low, with a correlation coefficient of 0.45; therefore, as suggested in O ’Donnell et al. [23], we use both measures of SES to test the robustness of our results. To report our main findings, however, household consumption was used as our preferred direct measure of SES. A detailed description of the construction of SES indicators is presented in the Technical Appendix.

### Measuring SES gradients

SES gradients in child development were evaluated by comparing development outcomes across five quintiles of the population ranked by the level of consumption or wealth index. Quintiles were constructed based on the distribution of the household population rather than on the distribution of households; therefore, Quintile 1 (Q1) corresponds to children in the poorest 20% of the population, whereas Quintile 5 (Q5) to children in the richest 20%^15^. The same classification of quintiles was used for the analysis of all child development outcomes. Given its straightforward interpretation, comparisons of outcomes by quintile have been widely used to characterize gradients in child’s health and development and are considered a preferable approach when other more complex measures of inequality do not provide additional insight of the problem [28].

In addition to the descriptive approach, we used a regression framework to assess the adjusted associations between child development outcomes and socioeconomic status, after controlling for other demographic factors that might affect child development. The SES gap in child development was estimated by OLS using the following equation:

1 $$ {Y}_i=\alpha +{\sum}_{k=2}^5{\beta}_k{Q}_{ki}+\gamma {age}_i+\delta {female}_i+{\varepsilon}_i $$

where *Y*_*i*_ is the development outcome for child *i; age*_*i*_ are semi-parametric controls for child’s age in months using 3-month bins; *female*_*i*_ is a dummy variable equal one if child is a girl and zero otherwise. *Q*_*ki*_ is a binary indicator for the *k*th quintile of the SES distribution where the omitted category is *Q*_1_. Hence, the estimated coefficient ${\hat{\beta}}_k$ represents the difference in the average outcome obtained by children in quintile *k* with respect to the average outcome in *Q*_1_. Joint hypothesis testing was used to test the association between the outcome and socioeconomic status across all quintiles^16^. In all analytical approaches, survey sampling design, including sample weights and clustering effects, were considered when computing point estimates and standard errors.

## Results

### Descriptive overview

This section presents descriptive statistics and unadjusted mean differences between high and low consumption quintiles for our outcomes of interest. For each measure, Table 1 shows mean, standard error and sample size for the whole sample of children and disaggregated for children at the bottom quintile (Q1) and the top quintile (Q5) of household consumption. The last two columns show the unadjusted difference in means between high and low quintile children and the *p*-value for the test of equality of means.

**Table 1:** Estimated means and standard errors by SES

**Indicator**	**All**			**Poorest 20% (Q1)**			**Richest 20% (Q5)**			**Q5-Q1**	
	**Mean**	**SE**	**N**	**Mean**	**SE**	**N**	**Mean**	**SE**	**N**	**diff**	***p*-value**
Height for age z-score (HAZ)	-0.979	0.024	10,870	-1.528	0.036	3,895	-0.577	0.049	1,334	0.952	0.000
Weight for age z-score (WAZ)	-0.185	0.021	10,469	-0.582	0.034	3,665	0.114	0.044	1,321	0.696	0.000
Weight for height z-score (WHZ)	0.523	0.022	10,453	0.411	0.034	3,660	0.624	0.043	1,317	0.213	0.000
Stunting (%) (HAZ<-2SD)	0.181	0.007	10,870	0.333	0.013	3,895	0.099	0.010	1,334	-0.234	0.000
Underweight (%) (WAZ<-2SD)	0.016	0.002	10,453	0.025	0.004	3,660	0.010	0.003	1,317	-0.015	0.007
Overweight (%) (WHZ>+2SD)	0.074	0.004	10,453	0.057	0.006	3,660	0.101	0.010	1,317	0.045	0.000
Anemia (%)	0.540	0.010	9,414	0.686	0.016	3,260	0.409	0.017	1,157	-0.278	0.000
RBP level (mmol/l)	0.943	0.105	1,609	1.040	0.238	516	1.032	0.130	216	-0.009	0.957
sTfR level (mg/l)	7.965	0.239	1,609	8.247	0.518	516	7.407	0.438	216	-0.840	0.226
Vitamin A deficiency (%) (<0.7 mmol/l)	0.391	0.023	1,609	0.390	0.031	516	0.299	0.035	216	-0.091	0.044
Iron deficiency (%) (>8.3mg/l)	0.240	0.017	1,609	0.238	0.030	516	0.164	0.027	216	-0.074	0.062
Gross motor z-score	-0.001	0.032	5,753	-0.061	0.056	2,031	0.151	0.047	680	0.212	0.003
Communication z-score	-0.005	0.031	5,753	0.007	0.049	2,031	0.115	0.057	680	0.108	0.160

Notes: Data are from the ESNUT 2012. Means and standard errors estimated considering survey sampling design, including sample weights and clustering effects.

There were large and statistically significant differences in all anthropometrics z-scores between children in Q5 and children in Q1: height for age (0.95 SD), weight for age (0.70 SD), and weight for height (0.21 SD). Differences were also demonstrated for prevalence of stunting: while one in every ten children were stunted in the richest quintile, this proportion more than tripled to one in every three children in the poorest quintile. Although the prevalence of underweight is low in Bolivia, the percentage of poor children that were underweight (2.5%) more than doubled that of the richest group. On the other hand, the prevalence of overweight for children in the top quintile (10.1%) almost doubled that of children at the bottom quintile (5.7%).

Results for biomarkers showed that the prevalence of anemia in Bolivia was high, with more than half of children (54%) suffering from anemia in 2012. When dividing the sample in children at the bottom and top consumption quintiles the results showed that 68.6% of children in the poorest quintile were anemic, compared to 40.9% in the richest quintile. Interestingly, although anemia is significantly lower among the richest group, it is still higher than average values in most other countries in the region. Similarly, the prevalence of vitamin A deficiency was 29.9% for children in the richest quintile and almost 10 percentage points higher for those in the poorest quintile (39.0%). By contrast, the SES gap in iron deficiency was somewhat smaller with prevalence varying from 16.4% in the richest group to 23.8% in the poorest group. The socioeconomic gaps for these indicators were all statistically significant at the 5% confidence level, except for iron deficiency that was significant at the 10% level.

Finally, Table 1 shows mean standardized gross motor and communication z-scores which are centered at zero since they were constructed within sample. The results showed that children in the richest quintile have a gross motor score 0.21 SD higher than children in the poorest quintile. For the communication z-scores, the difference between children in the poorest and richest quintiles was 0.11 SD, but not statistically significant. Overall, the descriptive analysis indicates that there were large and significant gaps in child development and its associated nutritional risk factors by socioeconomic status.

### Non-Parametric relation of SES gap and age

**Fig. 1:**
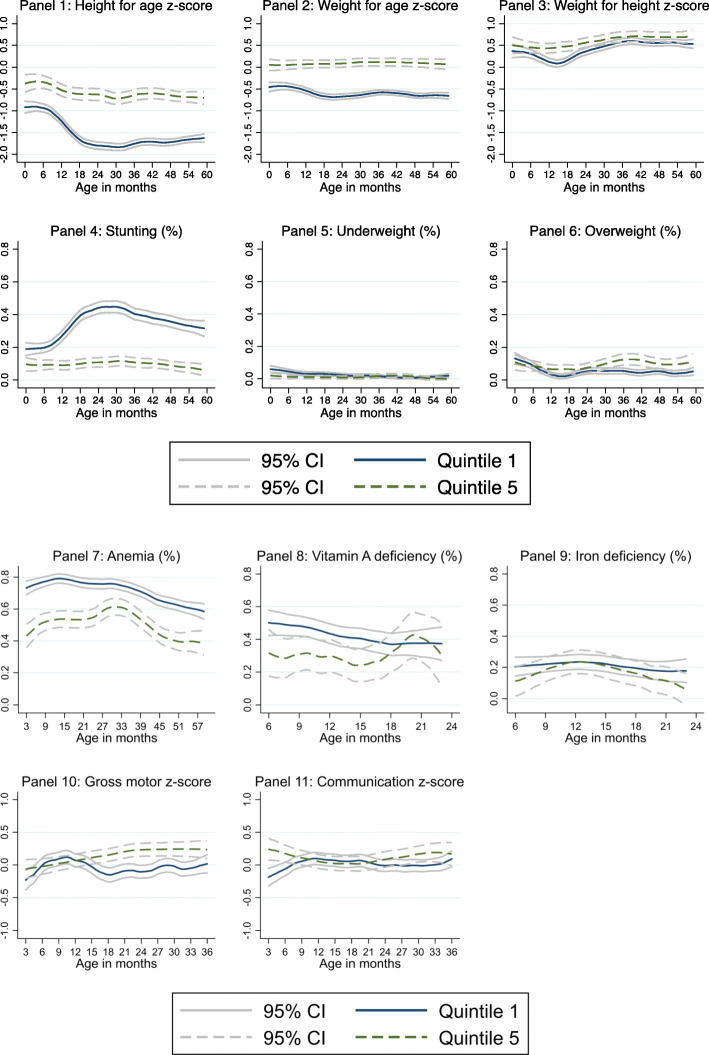
Child nutrition and development indicators by consumption quintile across age cohorts

To explore the onset and evolution of the socioeconomic gap in child development, we analyzed the differences in outcomes between the bottom and top quintiles across different age groups. In Fig. 1 we plotted kernel weighted local polynomial regressions of standardized scores for all child nutrition and development indicators and reported 95% confidence intervals for each group.

The first panel shows that even at very young ages there was a significant SES gap in height for age, and that this gap widened for children in older age groups. In the first five months of life, the gap was approximately 0.5 SD. Among older children, the z-score of height for age decreased for children in both the poorest and richest quintiles; however, the gap between them started to broaden markedly at 6 months until it reached its maximum at 24 months, stabilizing at around 1 SD. The gap path over age is similar when we analyze prevalence of stunting (Panel 4). At the first five months children in the poorest households were approximately 7 percentage points more likely to be stunted than those in the richest households. This gap reached a noticeable peak of more than 25 percentage points for 24-month-old children. While the gap tends to decrease slightly for older cohorts, it remained at 20 percentage points among five-year-old children.

The next panels show weight for age and weight for height z-scores as well as prevalence of children underweight and overweight. In the case of weight for age (Panel 2), the socioeconomic gap widened from approximately 0.5 SD during the first 6 months of age to 0.65 SD at 18 months, stabilizing around this number at older ages. For prevalence of underweight (Panel 5) the evidence suggests that any initial (non-significant) gap between children in the top and bottom quintiles tends to disappear with age. The gap between rich and poor children in the weight for height z-score showed that while it tended to increase significantly from birth until 15 months of age, it reduced again in the following two years (Panel 3). Finally, while differences in prevalence of overweight between the richest and poorest children were less clear during the first two years of life, the gap increased and became more relevant among three-year-olds, remaining relatively stable at approximately 6 percentage points (Panel 6).

In Panels 7 to 9 we analyzed the SES gap of nutrition indicators obtained from biomarkers. Panel 7 shows how the gap in anemia between children in the richest and poorest quintile changed by age group. Noticeably, the largest SES gap in anemia occurred in early infancy (around 30 percentage points), declined for older cohorts and tended to increase again among children between three and five years of age. Panels 8 and 9 present vitamin A deficiency and iron deficiency by age, respectively. Since DBS were taken only for a random sample of children aged 6 to 24 months, confidence intervals for indicators based on DBS were substantially larger. The results for VAD showed a difference of approximately 20 percentage points between the poorest and richest quintile for children 6 to 9 months old; however, the gap closed rapidly at older ages. The prevalence of iron deficiency showed no significant differences between poorest and richest children when we compared within age groups.

The last two panels of Fig. 1 show socioeconomic differences in standardized scores of gross motor and communication skills across age groups. The gap in gross motor skills became positive around the age of 15 months, stabilizing at about 0.30 SD in favor of the richest group. For communication skills, results showed no significant differences between children in Q1 and Q5 within age groups, although for some multivariate regression models discussed below the SES gaps in communication become apparent.

Overall, the nonparametric analysis of gaps within age subgroups showed that, for key nutritional indicators, there were significant SES gaps that started early in life (e.g. anemia, stunting). In some cases, the gaps increased markedly for older children (e.g. stunting) and in other cases they remained relatively stable over time. The gap, while it exists, was less noticeable for measures of vitamin A and iron deficiency.

### Parametric estimation of SES gaps in child development

Results of parametric regressions defined in equation (1) for the whole sample and disaggregated by ecological regions are presented in Table 2. After adjusting for sex and age, there was a strong and significant association between SES and almost every anthropometric indicator. Compared to children in the poorest quintile Q1, children were 0.30 SD and 0.65 SD taller for their age in Q2 and Q3, respectively. This gap increases to 0.96 SD for children in the richest quintile. The results followed a similar SES gradient for weight for age and weight for height z-scores, where higher quintiles were associated with higher z-scores. The results for prevalence of stunting showed that 30% of children in Q1 were stunted, after adjusting for age and sex. This number reduced by one third (11.2 percentage points) for children in the next consumption quintile and decreased even more markedly across higher quintiles. We found a similar pattern of inequality for the prevalence of underweight. Children in Q5 had a 21% lower probability of being underweight than children in Q1. In the case of prevalence of overweight, children in the richest quintile were 4.6 percentage points more likely to be overweight than children in the poorest quintile, a relative difference of 37%.

**Table 2:** Adjusted SES gradients in child nutrition and development indicators by region

	**Height for age z-score**				**Weight for age z-score**				**Weight for height z-score**			
VARIABLES	All	Highlands	Valleys	Lowlands	All	Highlands	Valleys	Lowlands	All	Highlands	Valleys	Lowlands
Quintile 2 = 1	0.297*** (0.052)	0.252*** (0.084)	0.220*** (0.077)	0.291*** (0.088)	0.229*** (0.045)	0.193** (0.079)	0.268*** (0.071)	0.146** (0.072)	0.073 (0.045)	0.085 (0.065)	0.177** (0.078)	-0.044 (0.094)
Quintile 3 = 1	0.647*** (0.051)	0.451*** (0.080)	0.656*** (0.071)	0.613*** (0.086)	0.451*** (0.049)	0.331*** (0.081)	0.501*** (0.084)	0.382*** (0.076)	0.110** (0.050)	0.095 (0.079)	0.197** (0.078)	0.027 (0.099)
Quintile 4 = 1	0.780*** (0.058)	0.526*** (0.107)	0.684*** (0.084)	0.775*** (0.086)	0.541*** (0.050)	0.450*** (0.088)	0.409*** (0.080)	0.556*** (0.087)	0.162*** (0.049)	0.265** (0.104)	0.056 (0.071)	0.168 (0.103)
Quintile 5 = 1	0.962*** (0.061)	0.722*** (0.096)	0.819*** (0.079)	1.014*** (0.107)	0.697*** (0.058)	0.555*** (0.126)	0.563*** (0.087)	0.746*** (0.096)	0.214*** (0.055)	0.182 (0.141)	0.143 (0.086)	0.253** (0.101)
Female	0.099*** (0.034)	0.179*** (0.052)	0.122** (0.056)	0.014 (0.058)	0.075** (0.029)	0.220*** (0.043)	0.064 (0.047)	-0.018 (0.056)	0.033 (0.030)	0.169*** (0.053)	0.009 (0.046)	-0.037 (0.056)
Constant	-1.281*** (0.098)	-1.534*** (0.189)	-1.433*** (0.153)	-0.706*** (0.133)	-0.843*** (0.096)	-1.114*** (0.138)	-0.891*** (0.151)	-0.433** (0.169)	0.132 (0.121)	0.110 (0.148)	0.256 (0.172)	0.030 (0.292)
Observations	10,870	3,446	3,701	3,723	10,469	3,404	3,395	3,670	10,453	3,397	3,391	3,665
R-squared	0.132	0.094	0.115	0.157	0.067	0.071	0.051	0.076	0.020	0.038	0.017	0.044
*p-*value (F-test for quintile effects)	0.0000	0.0000	0.0000	0.0000	0.0000	0.0000	0.0000	0.0000	0.0020	0.1649	0.0728	0.0146
	**Stunting (%)**				**Underweight (%)**				**Overweight (%)**			
VARIABLES	All	Highlands	Valleys	Lowlands	All	Highlands	Valleys	Lowlands	All	Highlands	Valleys	Lowlands
Quintile 2 = 1	-0.112*** (0.018)	-0.143*** (0.030)	-0.077*** (0.029)	-0.070** (0.032)	-0.009 (0.006)	-0.012 (0.009)	-0.017* (0.009)	-0.003 (0.013)	0.005 (0.009)	0.009 (0.014)	0.007 (0.016)	-0.010 (0.017)
Quintile 3 = 1	-0.184*** (0.016)	-0.198*** (0.031)	-0.186*** (0.025)	-0.120*** (0.026)	-0.003 (0.005)	-0.004 (0.010)	-0.002 (0.010)	-0.005 (0.009)	0.014 (0.010)	0.016 (0.018)	0.022 (0.017)	-0.009 (0.021)
Quintile 4 = 1	-0.216*** (0.017)	-0.201*** (0.037)	-0.206*** (0.025)	-0.161*** (0.029)	-0.016*** (0.005)	-0.020*** (0.007)	-0.006 (0.010)	-0.022*** (0.008)	0.023** (0.011)	0.049** (0.024)	-0.003 (0.016)	0.017 (0.022)
Quintile 5 = 1	-0.236*** (0.016)	-0.260*** (0.034)	-0.229*** (0.022)	-0.163*** (0.030)	-0.013** (0.005)	-0.013 (0.012)	-0.017** (0.009)	-0.012 (0.009)	0.046*** (0.012)	0.049 (0.032)	0.011 (0.016)	0.061** (0.025)
Female	-0.034*** (0.011)	-0.060** (0.024)	-0.041** (0.018)	-0.008 (0.016)	-0.012*** (0.003)	-0.018*** (0.006)	-0.012* (0.006)	-0.010 (0.006)	-0.013* (0.008)	0.011 (0.011)	-0.020 (0.012)	-0.026** (0.013)
Constant	0.295*** (0.026)	0.310*** (0.049)	0.345*** (0.044)	0.169*** (0.036)	0.075*** (0.016)	0.052** (0.021)	0.089*** (0.031)	0.076*** (0.025)	0.119*** (0.029)	0.093** (0.043)	0.136*** (0.033)	0.136* (0.072)
Observations	10,870	3,446	3,701	3,723	10,453	3,397	3,391	3,665	10,453	3,397	3,391	3,665
R-squared	0.067	0.074	0.073	0.045	0.014	0.022	0.022	0.021	0.011	0.025	0.013	0.027
*p-*value (F-test for quintile effects)	0.0000	0.0000	0.0000	0.0000	0.0164	0.0083	0.2071	0.0008	0.0051	0.3072	0.5655	0.0097
	**Anemia (%)**				**VAD (%)**				**Iron deficiency (%)**			
VARIABLES	All	Highlands	Valleys	Lowlands	All	Highlands	Valleys	Lowlands	All	Highlands	Valleys	Lowlands
Quintile 2 = 1	-0.103*** (0.020)	-0.144*** (0.031)	-0.046 (0.032)	-0.080** (0.036)	0.067 (0.055)	-0.122 (0.085)	0.216** (0.090)	0.134 (0.113)	0.029 (0.048)	0.095 (0.077)	0.032 (0.074)	-0.074 (0.086)
Quintile 3 = 1	-0.164*** (0.023)	-0.167*** (0.044)	-0.092** (0.036)	-0.161*** (0.033)	0.073 (0.049)	-0.141** (0.063)	0.068 (0.077)	0.177* (0.100)	0.020 (0.043)	0.103 (0.094)	0.097 (0.063)	-0.081 (0.078)
Quintile 4 = 1	-0.186*** (0.026)	-0.097** (0.047)	-0.171*** (0.036)	-0.152*** (0.045)	-0.023 (0.064)	-0.184** (0.091)	-0.106* (0.061)	0.050 (0.126)	0.019 (0.047)	0.322*** (0.109)	0.090 (0.060)	-0.105 (0.090)
Quintile 5 = 1	-0.286*** (0.022)	-0.334*** (0.044)	-0.206*** (0.033)	-0.244*** (0.031)	-0.085* (0.044)	-0.144* (0.081)	-0.070 (0.061)	-0.077 (0.108)	-0.084** (0.038)	-0.017 (0.077)	-0.052 (0.056)	-0.099 (0.090)
female	-0.039*** 0.015)	-0.058* (0.032)	-0.045* (0.023)	-0.014 (0.022)	-0.012 (0.029)	-0.017 (0.050)	-0.017 (0.039)	-0.009 (0.056)	-0.109*** (0.029)	-0.150*** (0.050)	-0.122*** (0.044)	-0.069 (0.047)
Constant	0.621*** (0.037)	0.802*** (0.070)	0.543*** (0.057)	0.526*** (0.058)	0.466*** (0.045)	0.601*** (0.088)	0.353*** (0.064)	0.547*** (0.102)	0.229*** (0.040)	0.358*** (0.075)	0.148** (0.057)	0.216** (0.095)
Observations	9,414	2,894	3,205	3,315	1,609	548	535	526	1,609	548	535	526
R-squared	0.097	0.096	0.110	0.101	0.034	0.086	0.060	0.072	0.046	0.091	0.090	0.046
*p-*value (F-test for quintile effects)	0.0000	0.0000	0.0000	0.0000	0.0079	0.1262	0.0062	0.0149	0.0239	0.0011	0.0499	0.8080
	**Gross motor z-score**					**Communication z-score**						
VARIABLES	All	Highlands	Valleys	Lowlands		All	Highlands		Valleys		Lowlands	
Quintile 2 = 1	0.028 (0.072)	0.035 (0.085)	-0.019 (0.086)	0.066 (0.227)		-0.133* (0.071)	-0.171** (0.082)		-0.101 (0.106)		-0.092 (0.176)	
Quintile 3 = 1	0.016 (0.061)	-0.000 (0.122)	-0.099 (0.079)	0.106 (0.150)		0.037 (0.059)	0.042 (0.106)		0.039 (0.100)		0.056 (0.122)	
Quintile 4 = 1	0.041 (0.069)	-0.078 (0.161)	-0.081 (0.112)	0.126 (0.144)		-0.084 (0.067)	-0.038 (0.149)		-0.101 (0.110)		-0.112 (0.113)	
Quintile 5 = 1	0.210*** (0.070)	-0.015 (0.149)	0.051 (0.090)	0.367* (0.197)		0.113 (0.073)	-0.015 (0.136)		0.126 (0.108)		0.159 (0.156)	
Female	-0.076* (0.040)	-0.059 (0.066)	-0.080 (0.057)	-0.088 (0.077)		0.091*** (0.035)	0.098 (0.060)		0.075 (0.062)		0.110* (0.063)	
Constant	-0.114 (0.076)	-0.053 (0.135)	-0.102 (0.104)	-0.159 (0.206)		0.098 (0.078)	-0.019 (0.129)		0.099 (0.139)		0.159 (0.140)	
Observations	5,753	1,909	1,912	1,932		5,753	1,909		1,912		1,932	
R-squared	0.015	0.027	0.033	0.025		0.016	0.017		0.031		0.048	
*p-*value (F-test for quintile effects)	0.0076	0.9613	0.5239	0.0039		0.0082	0.2081		0.3110		0.1033	

Notes: ***significant at the 1% level; ** significant at the 5% level; *significant at the 10% level. SE clustered at the PSU level (census sector or segment) in parentheses. OLS Estimation. Controls include child’s sex and a set of child dummies for age categories in months (0-2, 3-5, 6-8, 9-11, 12-14, 15-17, 18-20, 21-23, 24-26, 27-29, 30-32, 33-35, 36-38, 39-41, 42-44, 45-47, 48-50, 51-53, 54-56, 57-59). Quintiles are population quintiles using monthly per capita household consumption as the ranking variable. *p*-value (*F*-test for quintile effects) is the *p*-value of the *F*-test of Q2=Q3=Q4=Q5=0.

Our results also showed a clear SES gradient in prevalence of anemia. When compared to children in the poorest quintile, anemia was 10.3 and 16.4 percentage points lower in Q2 and Q3, respectively. Among children in the richest quintile, the prevalence dropped by 28.6 percentage points (compared to Q1). For vitamin A deficiency, iron deficiency and gross motor development the association with socioeconomic status was less strong. Although the percentage of children with deficits in vitamin A or iron did not differ for children in Q2-Q4, relative to children in Q1, it reduced significantly for children in Q5 (8.5 and 8.4 percentage points for vitamin A and iron, respectively). Similarly, gross motor z-score among children in the richest quintile was 0.21 SD higher compared to children in the poorest quintile.

Table 2 also presents adjusted SES gaps for ecological regions. While for some indicators SES gradients were strongly significant in all regions (e.g. height for age, weight for age, stunting, underweight and anemia), other indicators presented mixed results. Notably, there were significant SES gaps in the prevalence of overweight children in the highlands (Q4 vs Q1) and lowlands (Q5 vs Q1), but not in the valleys. Socioeconomic related inequalities in vitamin A deficiency were more evident in the highlands than in the valleys or lowlands.

Table 3 presents estimated coefficients disaggregating the sample by age subgroups. The results for height for age, weight for age, and prevalence of stunting showed that the SES gradient reached its peak in children between 24 and 36 months . Prevalence of stunting was 9.4 percentage points lower for children in Q5 with respect to Q1 in the first 3-11 months of age, whereas this difference became 29.5 percentage points for children aged 24 to 36 months. Because stunting is an indicator of chronic malnutrition and is affected by multidimensional factors, this pattern could be the result of children in poor families being exposed to multiple and cumulative hazards as they grow. This pattern was somewhat different for anemia where SES gaps were largest in children less than one and at four years of age. For vitamin A and iron deficiency the sample was too small to efficiently estimate SES gaps within age groups.

**Table 3:** Adjusted SES gradients in child nutrition and development indicators by child’s age

	**Height for age z-score**				**Weight for age z-score**				**Weight for height z-score**			
VARIABLES	3-11 Months	12-23 Months	24-36 Months	37-59 Months	3-11 Months	12-23 Months	24-36 Months	37-59 Months	3-11 Months	12-23 Months	24-36 Months	37-59 Months
Quintile 2 = 1	0.133 (0.127)	0.288*** (0.095)	0.383*** (0.087)	0.313*** (0.069)	0.204* (0.106)	0.296*** (0.091)	0.267*** (0.080)	0.204*** (0.062)	0.165 (0.100)	0.186** (0.089)	0.097 (0.085)	0.025 (0.067)
Quintile 3 = 1	0.429*** (0.134)	0.617*** (0.091)	0.692*** (0.080)	0.749*** (0.069)	0.381*** (0.108)	0.538*** (0.087)	0.388*** (0.086)	0.508*** (0.066)	0.211* (0.112)	0.293*** (0.088)	0.015 (0.093)	0.079 (0.065)
Quintile 4 = 1	0.606*** (0.136)	0.821*** (0.104)	0.962*** (0.088)	0.768*** (0.074)	0.449*** (0.128)	0.561*** (0.100)	0.676*** (0.087)	0.538*** (0.061)	0.116 (0.129)	0.226** (0.103)	0.198** (0.090)	0.147** (0.063)
Quintile 5 = 1	0.608*** (0.146)	0.988*** (0.127)	1.124*** (0.099)	1.067*** (0.084)	0.521*** (0.131)	0.781*** (0.107)	0.739*** (0.086)	0.755*** (0.082)	0.241* (0.123)	0.390*** (0.108)	0.159* (0.086)	0.167** (0.076)
Female = 1	0.173** (0.084)	0.244*** (0.072)	0.157** (0.062)	-0.061 (0.053)	0.165** (0.074)	0.193*** (0.065)	0.111** (0.053)	-0.084 (0.052)	0.098 (0.073)	0.105* (0.061)	0.039 (0.054)	-0.051 (0.048)
Constant	-0.798*** (0.118)	-1.665*** (0.099)	-1.854*** (0.087)	-1.646*** (0.092)	-0.352*** (0.109)	-0.728*** (0.089)	-0.721*** (0.075)	-0.539*** (0.092)	0.280*** (0.104)	0.107 (0.092)	0.376*** (0.074)	0.553*** (0.092)
Observations	1,718	2,391	2,411	3,882	1,693	2,285	2,325	3,707	1,689	2,285	2,323	3,702
R-squared	0.045	0.097	0.125	0.129	0.034	0.071	0.080	0.077	0.008	0.023	0.009	0.008
*p*-value (*F-*test for quintile effects)	0.0000	0.0000	0.0000	0.0000	0.0001	0.0000	0.0000	0.0000	0.1991	0.0027	0.1407	0.0864
	**Stunting (%)**				**Underweight (%)**				**Overweight (%)**			
VARIABLES	3-11 Months	12-23 Months	24-36 Months	37-59 Months	3-11 Months	12-23 Months	24-36 Months	37-59 Months	3-11 Months	12-23 Months	24-36 Months	37-59 Months
Quintile 2 = 1	-0.061* (0.031)	-0.149*** (0.039)	-0.120*** (0.040)	-0.116*** (0.027)	-0.009 (0.014)	-0.033*** (0.012)	-0.009* (0.005)	0.008 (0.011)	0.002 (0.022)	0.022 (0.017)	0.014 (0.020)	0.004 (0.014)
Quintile 3 = 1	-0.103*** (0.030)	-0.207*** (0.034)	-0.225*** (0.036)	-0.189*** (0.026)	-0.000 (0.017)	-0.035*** (0.013)	0.003 (0.009)	0.006 (0.007)	0.019 (0.027)	0.023 (0.018)	0.006 (0.017)	0.017 (0.017)
Quintile 4 = 1	-0.128*** (0.029)	-0.261*** (0.034)	-0.260*** (0.033)	-0.224*** (0.026)	-0.027** (0.012)	-0.032** (0.014)	-0.009* (0.005)	-0.007 (0.004)	0.015 (0.025)	0.004 (0.014)	0.035 (0.022)	0.025 (0.018)
Quintile 5 = 1	-0.094*** (0.034)	-0.286*** (0.032)	-0.295*** (0.034)	-0.255*** (0.023)	-0.029** (0.011)	-0.030** (0.014)	-0.006 (0.007)	-0.000 (0.006)	0.015 (0.030)	0.061*** (0.023)	0.042* (0.023)	0.069*** (0.020)
Female = 1	-0.045** (0.020)	-0.090*** (0.024)	-0.060*** (0.023)	0.026 (0.018)	-0.035*** (0.010)	-0.007 (0.007)	-0.004 (0.004)	-0.008 (0.006)	0.006 (0.019)	-0.002 (0.014)	-0.021 (0.018)	-0.025* (0.013)
Constant	0.206*** (0.028)	0.407*** (0.032)	0.465*** (0.035)	0.327*** (0.030)	0.061*** (0.014)	0.040*** (0.014)	0.018** (0.007)	0.007 (0.005)	0.097*** (0.019)	0.036** (0.017)	0.050*** (0.019)	0.078*** (0.024)
Observations	1,718	2,391	2,411	3,882	1,689	2,285	2,323	3,702	1,689	2,285	2,323	3,702
R-squared	0.029	0.073	0.070	0.061	0.023	0.013	0.005	0.011	0.007	0.010	0.008	0.013
*p*-value (*F-*test for quintile effects)	0.0002	0.0000	0.0000	0.0000	0.0466	0.0913	0.2188	0.1251	0.9426	0.0539	0.2821	0.0149
	**Anemia**				**Vitamin A deficiency**				**Iron deficiency**			
VARIABLES	3-11 Months	12-23 Months	24-36 Months	37-59 Months	6-11 Months	12-23 Months			6-11 Months	12-23 Months		
Quintile 2 = 1	-0.062 (0.044)	-0.032 (0.036)	-0.118** (0.045)	-0.147*** (0.034)	0.067 (0.099)	0.068 (0.060)			0.048 (0.080)	0.015 (0.057)		
Quintile 3 = 1	-0.117** (0.049)	-0.058 (0.036)	-0.244*** (0.045)	-0.201*** (0.037)	0.057 (0.078)	0.085 (0.059)			0.026 (0.068)	0.015 (0.054)		
Quintile 4 = 1	-0.165*** (0.057)	-0.062 (0.043)	-0.216*** (0.047)	-0.250*** (0.036)	-0.054 (0.089)	-0.004 (0.072)			0.046 (0.070)	0.003 (0.067)		
Quintile 5 = 1	-0.298*** (0.052)	-0.236*** (0.044)	-0.258*** (0.051)	-0.324*** (0.035)	-0.118 (0.078)	-0.064 (0.054)			-0.093* (0.055)	-0.080 (0.052)		
Female = 1	-0.062* (0.036)	-0.032 (0.026)	0.002 (0.030)	-0.057** (0.026)	-0.021 (0.049)	-0.006 (0.039)			-0.109** (0.047)	-0.110*** (0.035)		
Constant	0.611*** (0.041)	0.829*** (0.038)	0.791*** (0.042)	0.726*** (0.042)	0.487*** (0.059)	0.283*** (0.054)			0.220*** (0.045)	0.347*** (0.057)		
Observations	1,488	2,167	2,179	3,580	600	1,009			600	1,009		
R-squared	0.094	0.037	0.050	0.067	0.021	0.019			0.057	0.041		
*p*-value (*F-*test for quintile effects)	0.0000	0.0000	0.0000	0.0000	0.2829	0.0565			0.1933	0.3113		
	**Gross motor z-score**				**Communication z-score**							
VARIABLES	3-11 Months	12-23 Months	24-36 Months		3-11 Months	12-23 Months		24-36 Months				
Quintile 2 = 1	-0.058 (0.108)	-0.067 (0.103)	0.179 (0.116)		-0.161 (0.115)	-0.293*** (0.090)		0.057 (0.097)				
Quintile 3 = 1	-0.066 (0.107)	-0.051 (0.087)	0.135 (0.121)		0.239** (0.098)	-0.259*** (0.085)		0.233** (0.090)				
Quintile 4 = 1	-0.060 (0.104)	0.002 (0.119)	0.146 (0.117)		0.006 (0.108)	-0.199* (0.104)		-0.025 (0.111)				
Quintile 5 = 1	-0.032 (0.111)	0.143 (0.098)	0.439*** (0.114)		0.221* (0.119)	-0.095 (0.088)		0.252** (0.125)				
Female = 1	-0.135** (0.062)	-0.035 (0.066)	-0.079 (0.069)		-0.003 (0.066)	0.148** (0.065)		0.091 (0.060)				
Constant	0.024 (0.089)	-0.066 (0.088)	-0.210* (0.113)		0.067 (0.091)	0.032 (0.091)		-0.229** (0.089)				
Observations	1,476	2,185	2,092		1,476	2,185		2,092				
R-squared	0.010	0.008	0.034		0.027	0.018		0.019				
*p*-value (*F-*test for quintile effects)	0.9680	0.2535	0.0016		0.0007	0.0019		0.0283				

Notes: ***significant at the 1% level; ** significant at the 5% level; *significant at the 10% level. SE clustered at the PSU level (census sector or segment) in parentheses. OLS Estimation. Controls include child’s sex and a set of child dummies for age categories in months (0-2, 3-5, 6-8, 9-11, 12-14, 15-17, 18-20, 21-23, 24-26, 27-29, 30-32, 33-35, 36-38, 39-41, 42-44, 45-47, 48-50, 51-53, 54-56, 57-59). Quintiles are population quintiles using monthly per capita household consumption as the ranking variable. *p*-value (*F*-test for quintile effects) is the *p*-value of the *F*-test of Q2=Q3=Q4=Q5=0.

Regarding gross motor skills, we found that differences between children in the poorest and richest quintiles became more evident among older children: while gross motor z-score did not vary by quintile at very young ages, the Q1-Q5 gap was large and statistically significant among children aged 24-36 months (0.44 SD).

Socioeconomic gaps in communication development have been largely analyzed in the literature [4, 5, 20]. While our estimated coefficient on communication z-score for Q5 in Table 2 was positive for the full sample, it was not statistically significant at conventional levels. In Table 3 we observe a heterogenous relationship, with large and significant SES gaps in communication for Q3 and Q5 (relative to Q1) for children in the 3-11- and 24-36-month categories, and a reverse association for intermediate quintiles in the 12-23-month group. However, the relationship between communication z-score and alternative measures of SES depicted in Table 4 showed large and statistically significant differences between Q1 and Q5. The lack of consistency in the communication dimension might be related to the choice of instrument used for this analysis (ASQ-2), which in previous studies presented low internal validity compared to the “gold standard” Bayley-III [21, 22]^17^.

**Table 4:** Estimated Q1-Q5 gap using alternative SES measures

	**Regression Coefficient for Q5**	
	Consumption	Wealth Index
Height for age z-score (HAZ)	0.962***	0.934***
Weight for age z-score (WAZ)	0.697***	0.665***
Weight for height z-score (WHZ)	0.214***	0.210***
Stunting (HAZ<-2SD)	-0.236***	-0.220***
Underweight (WAZ<-2SD)	-0.013**	-0.010*
Overweight (WHZ>+2SD)	0.046***	0.034***
Anemia (%)	-0.286***	-0.280***
Vitamin A deficiency (%)	-0.085*	-0.167***
Iron deficiency (%)	-0.084**	0.030
Gross motor (z-score)	0.210***	0.176**
Communication (z-score)	0.113	0.174**

Notes: ***significant at the 1% level; ** significant at the 5% level; *significant at the 10% level. OLS Estimation. Controls include child’s sex and a set of child dummies for age categories in months (0-2, 3-5, 6-8, 9-11, 12-14, 15-17, 18-20, 21-23, 24-26, 27-29, 30-32, 33-35, 36-38, 39-41, 42-44, 45-47, 48-50, 51-53, 54-56, 57-59).

Finally, we analyzed the association between SES and development outcomes controlling for nutritional status to partial out nutritional status in the association of SES and child development. Tables 5 and 6 present the estimated SES gradients in gross motor and communicative development, respectively. Model (1) estimates SES gaps without nutritional controls, whereas models (2) to (7) include various nutritional outcomes as covariates. In general, results show an expected association between poor nutritional status and lower gross motor and communication development. This association is particularly significant with indicators of height-for-age and weight-for-age. However, after including nutritional status as controls, the association between socioeconomic status and child gross motor development remained significant and of similar magnitude to the unadjusted models. The estimated SES gaps for communication development also remained similar before and after accounting for nutritional status in the model.

**Table 5:** Adjusted SES gradients in Gross Motor z-score controlling for nutritional status

	Model	Model	Model	Model	Model	Model	Model
VARIABLES	(1)	(2)	(3)	(4)	(5)	(6)	(7)
Quintile 2 = 1	0.028 (0.072)	-0.010 (0.071)	0.013 (0.073)	0.040 (0.074)	-0.002 (0.074)	0.041 (0.075)	0.002 (0.078)
Quintile 3 = 1	0.016 (0.061)	-0.073 (0.059)	-0.029 (0.061)	0.033 (0.062)	-0.042 (0.062)	0.036 (0.063)	-0.032 (0.064)
Quintile 4 = 1	0.041 (0.069)	-0.074 (0.069)	-0.016 (0.069)	0.053 (0.070)	-0.023 (0.070)	0.055 (0.071)	-0.009 (0.072)
Quintile 5 = 1	0.210*** (0.070)	0.084 (0.070)	0.136* (0.071)	0.216*** (0.072)	0.142* (0.074)	0.221*** (0.072)	0.188** (0.080)
female	-0.076* (0.040)	-0.105*** (0.039)	-0.100** (0.040)	-0.082** (0.040)	-0.099** (0.041)	-0.082** (0.041)	-0.070* (0.042)
Height for age z score (hfa)		0.129*** (0.017)					
Weight for age z-score (wfa)			0.127*** (0.020)				
Weight for height z-score (wfh)				0.030 (0.021)			
Stunted (hfa <2SD)					-0.261*** (0.058)		
Underweight (wfa < 2SD)						-0.137 (0.201)	
Anemia (any level)							-0.113** (0.050)
Constant	-0.114 (0.076)	0.003 (0.081)	-0.082 (0.080)	-0.140* (0.078)	-0.038 (0.081)	-0.124 (0.080)	-0.043 (0.091)
Observations	5,753	5,641	5,464	5,460	5,641	5,460	5,098
R-squared	0.015	0.036	0.030	0.016	0.023	0.015	0.018

Notes: ***significant at the 1% level; ** significant at the 5% level; *significant at the 10% level. SE clustered at the PSU level (census sector or segment) in parentheses. OLS Estimation. Additional controls include child’s sex and a set of child dummies for age categories in months (0-2, 3-5, 6-8, 9-11, 12-14, 15-17, 18-20, 21-23, 24-26, 27-29, 30-32, 33-35, 36-38, 39-41, 42-44, 45-47, 48-50, 51-53, 54-56, 57-59). Quintiles are population quintiles using monthly per capita household consumption as the ranking variable.

**Table 6:** Adjusted SES gradients in Communication z-score controlling for nutritional status

	Model	Model	Model	Model	Model	Model	Model
VARIABLES	(1)	(2)	(3)	(4)	(5)	(6)	(7)
Quintile 2 = 1	-0.133* (0.071)	-0.155** (0.072)	-0.134* (0.073)	-0.123* (0.073)	-0.155** (0.074)	-0.121* (0.073)	-0.167** (0.078)
Quintile 3 = 1	0.037 (0.059)	-0.013 (0.061)	0.039 (0.062)	0.061 (0.061)	-0.002 (0.061)	0.061 (0.061)	0.010 (0.062)
Quintile 4 = 1	-0.084 (0.067)	-0.156** (0.068)	-0.093 (0.069)	-0.065 (0.068)	-0.135** (0.068)	-0.065 (0.069)	-0.114* (0.068)
Quintile 5 = 1	0.113 (0.073)	0.033 (0.073)	0.097 (0.075)	0.131* (0.075)	0.057 (0.076)	0.130* (0.075)	0.093 (0.080)
female	0.091*** (0.035)	0.078** (0.036)	0.087** (0.036)	0.094*** (0.036)	0.079** (0.036)	0.095*** (0.037)	0.095*** (0.036)
Height for age z score (hfa)		0.073*** (0.017)					
Weight for age z-score (wfa)			0.044** (0.018)				
Weight for height z-score (wfh)				-0.017 (0.018)			
Stunted (hfa <2SD)					-0.183*** (0.051)		
Underweight (wfa < 2SD)						0.174 (0.174)	
Anemia (any level)							-0.049 (0.045)
Constant	0.098 (0.078)	0.164** (0.079)	0.093 (0.080)	0.084 (0.080)	0.151* (0.079)	0.071 (0.081)	0.151* (0.084)
Observations	5,753	5,641	5,464	5,460	5,641	5,460	5,098
R-squared	0.016	0.024	0.020	0.018	0.021	0.019	0.020

Notes: ***significant at the 1% level; ** significant at the 5% level; *significant at the 10% level. SE clustered at the PSU level (census sector or segment) in parentheses. OLS Estimation. Additional controls include child’s sex and a set of child dummies for age categories in months (0-2, 3-5, 6-8, 9-11, 12-14, 15-17, 18-20, 21-23, 24-26, 27-29, 30-32, 33-35, 36-38, 39-41, 42-44, 45-47, 48-50, 51-53, 54-56, 57-59). Quintiles are population quintiles using monthly per capita household consumption as the ranking variable.

#### Robustness checks

As a check of robustness, we re-estimated all our results using a wealth index as the measure of socioeconomic status. All the analyses produced similar results, which suggests that our findings were consistent across different measures of socioeconomic status. A summary of these results is presented in Table 4, and further detailed in the Web Appendix (Tables 9). We also ran all the analyses for the subsample of children with DBS data. The results were similar, although standard errors were larger due to reduced sample size. This suggests that the comparison of results across indicators was not biased due to sample composition. Additionally, we reweighted the sample for differences in missing data in our outcomes of interest since children for which we have information may be systematically different from those whose parents agree to health measures (Web Appendix Tables 7 and 8). Finally, we ran all our analysis using Inverse Probability Weighting to account for sample selection of missing data [29]. Results, shown in Web Appendix Table 10 and 11, remained similar and consistent to main results.

## Discussion

Developmental delays in early childhood have life-long consequences for an individual’s future health, school performance, productivity, earnings and wellbeing [8–10, 30, 31] and for a society at large [7]. Research shows that poverty is one of the most detrimental risk factors associated with a child’s health and development, with poorer children failing to reach their developmental potential [2, 20]. Some of the mediating factors contributing to this negative relationship are illness, nutritional deficiencies, low parental education and poor home environments [2, 21, 22, 32]. SES gradients in child health and development are present and extensively documented across countries. However, analyzing disparities within countries is particularly important as it provides information to identify, prioritize, implement and evaluate more efficient policies and interventions aimed at reducing disparities in child development.

Our study explored SES disparities in child nutrition and development outcomes in the context of Bolivia using a broadly representative sample of the population and analyzing a more comprehensive set of outcomes than considered in the literature to date. Measures of child development included gross motor and communicative development, whereas nutritional status was assessed using anthropometric measures and indicators of prevalence of anemia, vitamin A and iron deficiency. In our main analysis we used household consumption to measure household socioeconomic status, though results were robust to alternative SES measures.

We found large disparities in child nutrition indicators by socioeconomic status. A nonparametric analysis within age groups indicates that the gap between children from rich and poor families started very early in life (stunting, anemia). In measures of height-for-age and stunting, the SES gap tended to increase rapidly from the sixth month of age, reaching its peak at 24 months and stabilizing at older ages. Socioeconomic gaps were less noticeable for other nutrition outcomes including vitamin A and iron deficiencies. This results was to some extend unexpected as most anemia in countries with high prevalence is caused largely or in whole by iron deficiency [33], though our sample size and age range is more limited for these measures. The results for our early child development outcomes showed that the gap between children from families in the upper and lower consumption quintiles became apparent between 24 and 36 months when it was 0.44 standard deviations in gross motor and 0.25 standard deviations in communication z-scores.

The parametric analysis suggested that SES inequality persisted after adjusting for demographic factors that affect child development. The analysis disaggregated by subnational regions shed light on the heterogenous relationship between socioeconomic status and child development. While some indicators (height for age, weight for age, stunting, anemia) demonstrate common large and significant SES gaps in all regions, others show significant disparities in some regions and not in others (overweight, vitamin A deficiency).

This study presents some limitations. First, in the absence of exogenous variation in SES within the study population, the relationships estimated between SES and children’s nutrition and development indicators are considered associations, and not causal effects. Second, while the results presented in this study are largely consistent with existing studies from other countries, and the study sample is nationally representative for Bolivia, these findings cannot be directly extrapolated to other country contexts. Third, although this study includes a rich set of nutritional outcomes, a subset of those outcomes (namely Vitamin A and iron deficiency) are measured on a sub-sample of children in the 6-23 month age range, reducing the external validity and limiting statistical precision for these analysis relative to outcomes measured for the entire sample.

However, the richness of our data allows us to look at child development outcomes, including gross motor and communication development, as well as nutrition risk factors, such as undernutrition, anemia and micronutrient deficiencies, within the same representative sample of children. In addition, the survey collected novel data from dried blood samples to measure vitamin A and iron deficiencies. This is unusual for health surveys in developing countries and this study helps to motivate other countries to follow similar strategies.

Our study complements the existing body of literature that focuses on particular subpopulations of disadvantaged children [4] or analyzes SES gaps in child development across countries using a single SES indicator and measure of child development [5]. We show that SES gaps are consistent across different measurements of SES status of children using data that that allows estimating SES gap at the national level as well as in subpopulations of interest, such as urban and rural households. Furthermore, unlike other related studies we compare, within sample, the SES gradient across child development, anthropometric measures, biomarkers, and micronutrient deficiencies. With these data we show that the relationship between SES and child development remains strong and significant once we control for risk factors related to nutritional status, which shows that there is and important association between wealth or income and child cognition development independent of nutrition. Further research should assess other channels by which income affects child development, such as environmental factors or parental behavior.

The evidence provided in this study shows that although a large proportion of young children in Bolivia are affected by specific developmental risks (anemia affects around 50% of children and 39% have vitamin A deficiency), it’s the poorest children that face the greatest threats that compromise their development. These disparities are evident at birth and need to be addressed urgently to reduce developmental inequities. From a policy perspective, the large socioeconomic gaps in nutrition outcomes documented here reinforce the need to strengthen efforts that tackle the multiple causes of malnutrition for the poorest. On this topic, the country has been implementing nation scale programs to incentivize the use of preventive health services for children and women during pregnancy (Program Bono Juana Azurduy), and programs that contribute to the prevention and care of malnutrition through multisectoral actions (Food and Nutrition Multisectoral Program in the Life Cycle). Although these programs have some level of prioritization, their national scope and limited targeting reduce their effectiveness to close socioeconomic gaps. Interventions in other areas related to child development are scarce in Bolivia, as there is still a need for national and subnational governments to prioritize early childhood development in their programs of work. An important step forward was taken in 2014 with the implementation of a pilot ECD program that sought to improve early child development by strengthening child-stimulation practices at home (Program Grow Well to Live Well). The program’s impact evaluation reported positive effects on cognitive, communication and fin motor development of children in poor families in rural areas [34].

## Figures and Tables

**Fig. 2:**
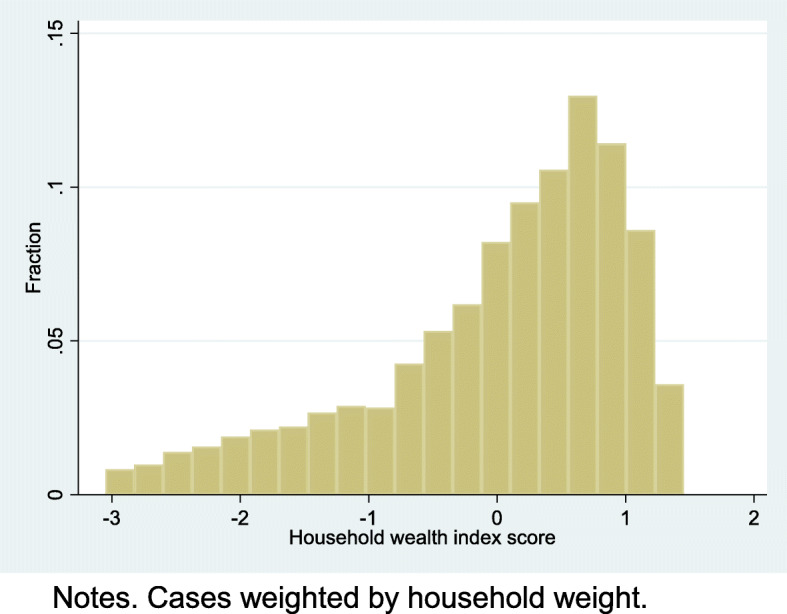
Distribution of households by wealth scores, Bolivia 2012. Notes. Cases weighted by household weight

## Data Availability

The data used in this study are available at http://ghdx.healthdata.org/record/bolivia-health-and-nutrition-assessment-survey-2012

## Appendix 1

### Web Appendix

**Table 7:** Adjusted SES gradients in child development by subnational region: DBS Subsample

	**Height for age z-score**				**Weight for age z-score**				**Weight for height z-score**			
VARIABLES	All	Highlands	Valleys	Lowlands	All	Highlands	Valleys	Lowlands	All	Highlands	Valleys	Lowlands
Quintile 2 = 1	0.162 (0.125)	-0.087 (0.168)	0.090 (0.170)	0.562** (0.255)	0.218* (0.111)	0.080 (0.177)	0.226 (0.168)	0.336* (0.189)	0.169 (0.107)	0.146 (0.174)	0.234 (0.185)	0.074 (0.177)
Quintile 3 = 1	0.481*** (0.121)	0.155 (0.179)	0.407** (0.195)	0.612** (0.240)	0.409*** (0.107)	0.354** (0.178)	0.462** (0.181)	0.250 (0.177)	0.203* (0.104)	0.340* (0.176)	0.333** (0.167)	-0.082 (0.167)
Quintile 4 = 1	0.742*** (0.132)	0.736 (0.469)	0.360** (0.162)	0.817*** (0.231)	0.478*** (0.112)	0.491 (0.335)	0.260 (0.178)	0.410** (0.182)	0.126 (0.113)	0.154 (0.273)	0.081 (0.194)	0.011 (0.169)
Quintile 5 = 1	0.847*** (0.131)	0.526* (0.267)	0.794*** (0.184)	0.911*** (0.259)	0.686*** (0.116)	0.567** (0.217)	0.644*** (0.213)	0.603*** (0.174)	0.329** (0.136)	0.367 (0.293)	0.302 (0.241)	0.216 (0.174)
Female	0.275*** (0.073)	0.314*** (0.112)	0.177* (0.097)	0.448*** (0.135)	0.160** (0.073)	0.207 (0.140)	0.101 (0.112)	0.246* (0.128)	0.023 (0.082)	0.044 (0.147)	0.026 (0.147)	0.017 (0.134)
Constant	-0.899*** (0.119)	-1.327*** (0.187)	-0.792*** (0.157)	-0.577** (0.259)	-0.397*** (0.108)	-0.624*** (0.196)	-0.191 (0.171)	-0.333* (0.196)	0.212* (0.120)	0.263 (0.216)	0.401** (0.199)	0.030 (0.194)
Observations	1,597	544	530	523	1,553	534	502	517	1,553	534	502	517
R-squared	0.154	0.118	0.150	0.237	0.073	0.074	0.077	0.086	0.012	0.034	0.024	0.035
	**Stunting (%)**				**Underweight (%)**				**Overweight (%)**			
VARIABLES	All	Highlands	Valleys	Lowlands	All	Highlands	Valleys	Lowlands	All	Highlands	Valleys	Lowlands
Quintile 2 = 1	-0.089** (0.044)	-0.089 (0.076)	-0.063 (0.072)	-0.140 (0.086)	-0.018 (0.018)	-0.022 (0.030)	-0.036 (0.025)	0.008 (0.024)	-0.012 (0.016)	0.002 (0.026)	-0.036 (0.027)	-0.013 (0.026)
Quintile 3 = 1	-0.164*** (0.036)	-0.221*** (0.063)	-0.113* (0.066)	-0.156** (0.067)	-0.030* (0.017)	-0.050* (0.026)	-0.021 (0.032)	-0.012 (0.016)	0.005 (0.017)	-0.003 (0.013)	0.010 (0.036)	-0.010 (0.029)
Quintile 4 = 1	-0.202*** (0.039)	-0.187 (0.115)	-0.146*** (0.055)	-0.223*** (0.076)	-0.026 (0.018)	-0.051** (0.025)	-0.006 (0.035)	-0.014 (0.016)	0.001 (0.018)	0.058 (0.053)	-0.020 (0.032)	-0.021 (0.026)
Quintile 5 = 1	-0.224*** (0.035)	-0.226*** (0.075)	-0.193*** (0.048)	-0.227*** (0.076)	-0.024 (0.018)	-0.049** (0.024)	-0.022 (0.031)	0.005 (0.027)	0.034 (0.024)	0.070 (0.057)	0.025 (0.041)	-0.001 (0.030)
Female	-0.089*** (0.023)	-0.079 (0.050)	-0.050 (0.038)	-0.139*** (0.038)	-0.008 (0.009)	-0.024 (0.018)	-0.003 (0.019)	-0.002 (0.009)	0.001 (0.014)	0.012 (0.022)	0.006 (0.029)	-0.015 (0.020)
Constant	0.261*** (0.033)	0.322*** (0.073)	0.188*** (0.041)	0.288*** (0.076)	0.053*** (0.019)	0.084** (0.042)	0.023 (0.026)	0.053* (0.029)	0.062** (0.024)	0.080* (0.046)	0.081* (0.046)	0.042 (0.028)
Observations	1,597	544	530	523	1,553	534	502	517	1,553	534	502	517
R-squared	0.104	0.099	0.098	0.136	0.011	0.047	0.020	0.039	0.012	0.064	0.012	0.026
	**Anemia (%)**				**VAD (%)**				**Iron deficiency (%)**			
VARIABLES	All	Highlands	Valleys	Lowlands	All	Highlands	Valleys	Lowlands	All	Highlands	Valleys	Lowlands
Quintile 2 = 1	-0.041 (0.045)	-0.122* (0.067)	0.088 (0.069)	0.056 (0.098)	0.067 (0.055)	-0.122 (0.085)	0.216** (0.090)	0.134 (0.113)	0.029 (0.048)	0.095 (0.077)	0.032 (0.074)	-0.074 (0.086)
Quintile 3 = 1	-0.025 (0.042)	-0.001 (0.056)	-0.048 (0.077)	0.109 (0.095)	0.073 (0.049)	-0.141** (0.063)	0.068 (0.077)	0.177* (0.100)	0.020 (0.043)	0.103 (0.094)	0.097 (0.063)	-0.081 (0.078)
Quintile 4 = 1	-0.096** (0.045)	0.041 (0.064)	-0.091 (0.067)	0.010 (0.105)	-0.023 (0.064)	-0.184** (0.091)	-0.106* (0.061)	0.050 (0.126)	0.019 (0.047)	0.322*** (0.109)	0.090 (0.060)	-0.105 (0.090)
Quintile 5 = 1	-0.182*** (0.049)	-0.114 (0.088)	-0.182*** (0.069)	-0.054 (0.109)	-0.085* (0.044)	-0.144* (0.081)	-0.070 (0.061)	-0.077 (0.108)	-0.084** (0.038)	-0.017 (0.077)	-0.052 (0.056)	-0.099 (0.090)
Female	-0.048* (0.028)	-0.063 (0.051)	-0.085* (0.051)	-0.015 (0.042)	-0.012 (0.029)	-0.017 (0.050)	-0.017 (0.039)	-0.009 (0.056)	-0.109*** (0.029)	-0.150*** (0.050)	-0.122*** (0.044)	-0.069 (0.047)
Constant	0.741*** (0.045)	0.816*** (0.077)	0.767*** (0.071)	0.539*** (0.106)	0.466*** (0.045)	0.601*** (0.088)	0.353*** (0.064)	0.547*** (0.102)	0.229*** (0.040)	0.358*** (0.075)	0.148** (0.057)	0.216** (0.095)
Observations	1,593	545	528	520	1,609	548	535	526	1,609	548	535	526
R-squared	0.036	0.062	0.089	0.021	0.034	0.086	0.060	0.072	0.046	0.091	0.090	0.046
	**Gross motor z-score**					**Communication z-score**						
VARIABLES	All	Highlands	Valleys	Lowlands		All	Highlands	Valleys	Lowlands			
Quintile 2 = 1	-0.168 (0.110)	-0.204 (0.168)	-0.057 (0.168)	-0.417* (0.240)		-0.302** (0.123)	-0.499*** (0.181)	-0.078 (0.186)	-0.409 (0.250)			
Quintile 3 = 1	-0.084 (0.106)	-0.133 (0.221)	-0.162 (0.165)	-0.189 (0.197)		-0.086 (0.111)	-0.350** (0.175)	0.017 (0.187)	-0.149 (0.197)			
Quintile 4 = 1	-0.072 (0.148)	-0.362 (0.293)	-0.246 (0.250)	-0.088 (0.271)		-0.152 (0.127)	-0.083 (0.252)	-0.117 (0.190)	-0.328 (0.222)			
Quintile 5 = 1	0.173* (0.100)	0.154 (0.208)	0.193 (0.162)	-0.097 (0.215)		-0.016 (0.124)	-0.098 (0.195)	0.203 (0.223)	-0.290 (0.204)			
Female	-0.107 (0.071)	0.007 (0.137)	-0.119 (0.109)	-0.183 (0.111)		0.105 (0.085)	0.174 (0.130)	0.079 (0.141)	0.097 (0.141)			
Constant	-0.020 (0.101)	-0.133 (0.165)	-0.036 (0.144)	0.280 (0.251)		0.147 (0.109)	-0.019 (0.190)	0.000 (0.160)	0.558** (0.214)			
Observations	1,473	515	489	469		1,473	515	489	469			
R-squared	0.021	0.031	0.044	0.051		0.022	0.056	0.020	0.062			

Note: ***significant at the 1% level; ** significant at the 5% level; *significant at the 10% level. SE clustered at the PSU level (census sector or segment) in parentheses. OLS Estimation. Controls include child’s sex and a set of child dummies for age categories in months (0-2, 3-5, 6-8, 9-11, 12-14, 15-17, 18-20, 21-23, 24-26, 27-29, 30-32, 33-35, 36-38, 39-41, 42-44, 45-47, 48-50, 51-53, 54-56, 57-59). Quintiles are population quintiles using monthly per capita household consumption as the ranking variable. *p*-value (*F*-test for quintile effects) is the *p*-value of the *F*-test of Q2=Q3=Q4=Q5=0.

**Table 8:** Adjusted SES gradients in child development by child’s age: DBS Subsample

	**Height for age z-score**		**Weight for age z-score**		**Weight for height z-score**		**Stunting (%)**		**Underweight (%)**		**Overweight (%)**	
VARIABLES	6-11 Months	12-23 Months	6-11 Months	12-23 Months	6-11 Months	12-23 Months	6-11 Months	12-23 Months	6-11 Months	12-23 Months	6-11 Months	12-23 Months
Quintile 2 = 1	0.126 (0.217)	0.199 (0.153)	0.068 (0.157)	0.343** (0.152)	0.003 (0.158)	0.305** (0.147)	-0.045 (0.051)	-0.125* (0.065)	0.012 (0.026)	-0.038 (0.026)	-0.056** (0.027)	0.018 (0.019)
Quintile 3 = 1	0.581*** (0.214)	0.458*** (0.131)	0.448*** (0.146)	0.434*** (0.136)	0.181 (0.142)	0.257* (0.138)	-0.127*** (0.031)	-0.197*** (0.051)	-0.016 (0.010)	-0.041 (0.027)	-0.052** (0.026)	0.039* (0.020)
Quintile 4 = 1	0.632*** (0.210)	0.817*** (0.163)	0.355** (0.179)	0.578*** (0.151)	0.027 (0.187)	0.214 (0.152)	-0.109*** (0.038)	-0.263*** (0.054)	-0.018* (0.009)	-0.033 (0.028)	0.012 (0.044)	0.000 (0.012)
Quintile 5 = 1	0.615*** (0.204)	0.991*** (0.153)	0.364** (0.159)	0.896*** (0.151)	0.033 (0.200)	0.526*** (0.174)	-0.125*** (0.035)	-0.290*** (0.051)	0.001 (0.022)	-0.041 (0.027)	-0.008 (0.043)	0.063** (0.028)
Female = 1	0.173 (0.130)	0.331*** (0.101)	0.001 (0.115)	0.253** (0.101)	-0.138 (0.123)	0.118 (0.104)	-0.036 (0.026)	-0.121*** (0.034)	-0.006 (0.014)	-0.011 (0.012)	-0.003 (0.025)	0.005 (0.017)
Constant	-0.777*** (0.144)	-1.614*** (0.138)	-0.185 (0.119)	-0.815*** (0.145)	0.428*** (0.131)	-0.027 (0.152)	0.176*** (0.030)	0.412*** (0.048)	0.036*** (0.013)	0.056* (0.031)	0.090*** (0.032)	0.029 (0.019)
Observations	598	999	589	964	589	964	598	999	589	964	589	964
R-squared	0.055	0.118	0.031	0.078	0.008	0.026	0.048	0.081	0.024	0.011	0.021	0.021
	**Anemia**		**VAD (%)**		**Iron deficiency (%)**		**Gross motor z-score**				**Communication z-score**	
VARIABLES	6-11 Months	12-23 Months	6-11 Months	12-23 Months	6-11 Months	12-23 Months	6-11 Months	12-23 Months			6-11 Months	12-23 Months
Quintile 2 = 1	-0.053 (0.070)	-0.035 (0.059)	0.067 (0.099)	0.068 (0.060)	0.048 (0.080)	0.015 (0.057)	-0.474*** (0.165)	0.044 (0.144)			-0.405** (0.205)	-0.220 (0.139)
Quintile 3 = 1	-0.064 (0.070)	-0.001 (0.058)	0.057 (0.078)	0.085 (0.059)	0.026 (0.068)	0.015 (0.054)	-0.261 (0.177)	0.042 (0.126)			0.073 (0.173)	-0.164 (0.131)
Quintile 4 = 1	-0.157* (0.082)	-0.059 (0.060)	-0.054 (0.089)	-0.004 (0.072)	0.046 (0.070)	0.003 (0.067)	-0.223 (0.177)	0.041 (0.183)			-0.074 (0.204)	-0.191 (0.158)
Quintile 5 = 1	-0.251*** (0.071)	-0.141** (0.068)	-0.118 (0.078)	-0.064 (0.054)	-0.093* (0.055)	-0.080 (0.052)	-0.087 (0.150)	0.344*** (0.130)			0.200 (0.200)	-0.133 (0.146)
Female = 1	-0.036 (0.062)	-0.055 (0.042)	-0.021 (0.049)	-0.006 (0.039)	-0.109** (0.047)	-0.110*** (0.035)	-0.157 (0.108)	-0.062 (0.092)			0.009 (0.130)	0.164 (0.101)
Constant	0.769*** (0.052)	0.831*** (0.052)	0.487*** (0.059)	0.283*** (0.054)	0.220*** (0.045)	0.347*** (0.057)	0.189 (0.121)	-0.210 (0.131)			0.137 (0.128)	0.010 (0.143)
Observations	598	995	600	1,009	600	1,009	556	917			556	917
R-squared	0.043	0.035	0.021	0.019	0.057	0.041	0.038	0.022			0.044	0.016

Note: ***significant at the 1% level; ** significant at the 5% level; *significant at the 10% level. SE clustered at the PSU level (census sector or segment) in parentheses. OLS Estimation. Controls include child’s sex and a set of child dummies for age categories in months (0-2, 3-5, 6-8, 9-11, 12-14, 15-17, 18-20, 21-23, 24-26, 27-29, 30-32, 33-35, 36-38, 39-41, 42-44, 45-47, 48-50, 51-53, 54-56, 57-59). Quintiles are population quintiles using monthly per capita household consumption as the ranking variable. *p*-value (*F*-test for quintile effects) is the *p*-value of the *F*-test of Q2=Q3=Q4=Q5=0.

**Table 9:** Adjusted SES gradients in child development using wealth index as measure of SES

VARIABLES	Height for age z-score	Weight for age z-score	Weight for height z-score	Stunting (%)	Underweight (%)	Overweight (%)	Anemia	VAD (%)	Iron deficiency (%)	Gross motor z-score	Commun. z-score
Quintile 2 = 1	0.244*** (0.056)	0.217*** (0.049)	0.104** (0.045)	-0.105*** (0.018)	-0.006 (0.005)	0.007 (0.009)	-0.075*** (0.022)	-0.102* (0.053)	0.098** (0.048)	-0.016 (0.078)	-0.008 (0.073)
Quintile 3 = 1	0.519*** (0.060)	0.392*** (0.051)	0.131** (0.057)	-0.172*** (0.019)	-0.013*** (0.004)	0.025** (0.011)	-0.157*** (0.024)	-0.117* (0.065)	0.039 (0.051)	0.150** (0.066)	0.081 (0.072)
Quintile 4 = 1	0.713*** (0.068)	0.530*** (0.054)	0.189*** (0.054)	-0.187*** (0.018)	-0.004 (0.006)	0.049*** (0.014)	-0.216*** (0.025)	-0.056 (0.053)	0.024 (0.044)	0.002 (0.074)	0.007 (0.074)
Quintile 5 = 1	0.934*** (0.067)	0.665*** (0.056)	0.210*** (0.057)	-0.220*** (0.017)	-0.010* (0.005)	0.034*** (0.012)	-0.280*** (0.026)	-0.167*** (0.053)	0.030 (0.043)	0.176** (0.079)	0.174** (0.080)
Female	0.110*** (0.034)	0.083*** (0.029)	0.035 (0.029)	-0.035*** (0.012)	-0.012*** (0.003)	-0.012 (0.008)	-0.042*** (0.015)	-0.011 (0.028)	-0.105*** (0.029)	-0.083** (0.039)	0.087** (0.035)
Constant	-1.202*** (0.101)	-0.805*** (0.098)	0.117 (0.123)	0.276*** (0.026)	0.073*** (0.016)	0.114*** (0.030)	0.612*** (0.033)	0.553*** (0.045)	0.184*** (0.041)	-0.105 (0.073)	0.048 (0.094)
Observations	10,884	10,481	10,466	10,884	10,466	10,466	9,427	1,610	1,610	5,761	5,761
R-squared	0.126	0.063	0.020	0.059	0.013	0.011	0.100	0.031	0.040	0.016	0.013

**Table 10:** Adjusted SES gradients in child development by region with IPW adjustments for missing data

	**Height for age z-score**				**Weight for age z-score**				**Weight for height z-score**			
VARIABLES	All	Highlands	Valleys	Lowlands	All	Highlands	Valleys	Lowlands	All	Highlands	Valleys	Lowlands
Quintile 2 = 1	0.298*** (0.054)	0.240*** (0.086)	0.209*** (0.075)	0.302*** (0.095)	0.237*** (0.046)	0.193** (0.081)	0.267*** (0.069)	0.167** (0.075)	0.080* (0.045)	0.083 (0.066)	0.184** (0.080)	-0.023 (0.096)
Quintile 3 = 1	0.626*** (0.052)	0.417*** (0.081)	0.608*** (0.071)	0.611*** (0.092)	0.449*** (0.049)	0.313*** (0.084)	0.486*** (0.079)	0.390*** (0.077)	0.113** (0.050)	0.080 (0.082)	0.198*** (0.076)	0.040 (0.104)
Quintile 4 = 1	0.766*** (0.058)	0.516*** (0.117)	0.659*** (0.085)	0.767*** (0.088)	0.539*** (0.052)	0.436*** (0.091)	0.406*** (0.082)	0.562*** (0.086)	0.160*** (0.049)	0.248** (0.105)	0.062 (0.073)	0.175 (0.108)
Quintile 5 = 1	0.941*** (0.063)	0.715*** (0.100)	0.772*** (0.084)	0.986*** (0.111)	0.691*** (0.059)	0.564*** (0.138)	0.544*** (0.088)	0.739*** (0.099)	0.212*** (0.057)	0.191 (0.148)	0.145 (0.089)	0.262** (0.108)
Female	0.119*** (0.035)	0.183*** (0.055)	0.130** (0.060)	0.040 (0.060)	0.085*** (0.030)	0.223*** (0.047)	0.063 (0.049)	-0.005 (0.056)	0.038 (0.030)	0.177*** (0.053)	0.012 (0.046)	-0.040 (0.054)
Constant	-1.278*** (0.053)	-1.599*** (0.092)	-1.327*** (0.075)	-0.800*** (0.097)	-0.573*** (0.048)	-0.781*** (0.081)	-0.565*** (0.074)	-0.304*** (0.094)	0.200*** (0.051)	0.159** (0.077)	0.247*** (0.075)	0.201 (0.126)
Observations	10,870	3,446	3,701	3,723	10,469	3,404	3,395	3,670	10,453	3,397	3,391	3,665
R-squared	0.102	0.069	0.080	0.113	0.056	0.054	0.037	0.057	0.015	0.020	0.013	0.029
	**Stunting (%)**				**Underweight (%)**				**Overweight (%)**			
VARIABLES	All	Highlands	Valleys	Lowlands	All	Highlands	Valleys	Lowlands	All	Highlands	Valleys	Lowlands
Quintile 2 = 1	-0.112*** (0.018)	-0.141*** (0.030)	-0.074*** (0.028)	-0.070** (0.033)	-0.010 (0.006)	-0.010 (0.010)	-0.017* (0.009)	-0.005 (0.013)	0.004 (0.009)	0.005 (0.014)	0.009 (0.016)	-0.008 (0.019)
Quintile 3 = 1	-0.181*** (0.016)	-0.190*** (0.031)	-0.175*** (0.024)	-0.118*** (0.026)	-0.004 (0.006)	-0.006 (0.010)	-0.001 (0.010)	-0.007 (0.010)	0.013 (0.010)	0.013 (0.017)	0.026 (0.017)	-0.009 (0.024)
Quintile 4 = 1	-0.213*** (0.017)	-0.196*** (0.037)	-0.200*** (0.025)	-0.158*** (0.030)	-0.018*** (0.005)	-0.022*** (0.007)	-0.008 (0.010)	-0.026*** (0.008)	0.020* (0.011)	0.048* (0.025)	-0.001 (0.016)	0.012 (0.025)
Quintile 5 = 1	-0.232*** (0.016)	-0.259*** (0.034)	-0.219*** (0.022)	-0.156*** (0.031)	-0.015*** (0.006)	-0.013 (0.011)	-0.018** (0.009)	-0.015 (0.010)	0.042*** (0.013)	0.050 (0.033)	0.011 (0.017)	0.055* (0.028)
Female	-0.039*** (0.011)	-0.067*** (0.025)	-0.043** (0.018)	-0.011 (0.016)	-0.012*** (0.003)	-0.017*** (0.005)	-0.012* (0.006)	-0.009 (0.006)	-0.011 (0.007)	0.012 (0.011)	-0.018 (0.012)	-0.023* (0.013)
Constant	0.323*** (0.016)	0.382*** (0.031)	0.340*** (0.022)	0.200*** (0.031)	0.042*** (0.006)	0.041*** (0.010)	0.049*** (0.011)	0.036*** (0.011)	0.067*** (0.011)	0.055*** (0.017)	0.087*** (0.015)	0.066** (0.031)
Observations	10,870	3,446	3,701	3,723	10,453	3,397	3,391	3,665	10,453	3,397	3,391	3,665
R-squared	0.052	0.054	0.052	0.031	0.008	0.010	0.012	0.007	0.004	0.009	0.004	0.012
	**Anemia (%)**				**VAD (%)**				**Iron deficiency (%)**			
VARIABLES	All	Highlands	Valleys	Lowlands	All	Highlands	Valleys	Lowlands	All	Highlands	Valleys	Lowlands
Quintile 2 = 1	-0.072*** (0.020)	-0.116*** (0.031)	-0.038 (0.032)	-0.060* (0.033)	0.082 (0.052)	-0.117 (0.084)	0.204** (0.080)	0.111 (0.110)	0.024 (0.047)	0.102 (0.078)	0.018 (0.070)	-0.094 (0.092)
Quintile 3 = 1	-0.149*** (0.024)	-0.139*** (0.046)	-0.073** (0.036)	-0.197*** (0.036)	0.060 (0.052)	-0.158** (0.068)	0.025 (0.077)	0.158 (0.104)	0.047 (0.042)	0.104 (0.085)	0.135** (0.065)	-0.065 (0.078)
Quintile 4 = 1	-0.170*** (0.025)	-0.084** (0.042)	-0.137*** (0.035)	-0.190*** (0.042)	-0.022 (0.063)	-0.183** (0.092)	-0.105* (0.060)	0.036 (0.126)	0.047 (0.050)	0.418*** (0.107)	0.088 (0.067)	-0.098 (0.095)
Quintile 5 = 1	-0.278*** (0.023)	-0.267*** (0.049)	-0.232*** (0.032)	-0.272*** (0.038)	-0.080* (0.045)	-0.182** (0.083)	-0.058 (0.061)	-0.096 (0.105)	-0.059 (0.037)	-0.022 (0.069)	-0.022 (0.053)	-0.098 (0.092)
Female	-0.054*** (0.014)	-0.085*** (0.029)	-0.057*** (0.021)	-0.028 (0.023)	-0.001 (0.030)	0.011 (0.050)	-0.005 (0.039)	-0.006 (0.059)	-0.110*** (0.031)	-0.148*** (0.055)	-0.132*** (0.043)	-0.058 (0.053)
Constant	0.813*** (0.021)	0.907*** (0.032)	0.783*** (0.029)	0.749*** (0.039)	0.454*** (0.059)	0.611*** (0.104)	0.385*** (0.082)	0.504*** (0.133)	0.349*** (0.049)	0.465*** (0.088)	0.353*** (0.070)	0.270*** (0.100)
Observations	9,414	2,894	3,205	3,315	1,590	542	530	518	1,590	542	530	518
R-squared	0.042	0.045	0.040	0.034	0.017	0.039	0.046	0.034	0.028	0.111	0.051	0.011
	**Gross motor z-score**				**Communication z-score**							
VARIABLES	All	Highlands	Valleys	Lowlands	All		Highlands	Valleys	Lowlands			
Quintile 2 = 1	-0.008 (0.070)	0.023 (0.103)	-0.069 (0.092)	-0.015 (0.186)	-0.166** (0.074)		-0.178 (0.108)	-0.156 (0.108)	-0.169 (0.173)			
Quintile 3 = 1	-0.007 (0.066)	0.027 (0.141)	-0.145 (0.089)	0.013 (0.111)	0.041 (0.063)		-0.031 (0.117)	0.012 (0.104)	0.078 (0.114)			
Quintile 4 = 1	0.006 (0.071)	-0.025 (0.142)	-0.179 (0.121)	0.039 (0.109)	-0.083 (0.071)		0.012 (0.126)	-0.155 (0.109)	-0.129 (0.119)			
Quintile 5 = 1	0.110 (0.073)	-0.014 (0.173)	-0.033 (0.101)	0.165 (0.150)	0.099 (0.078)		0.051 (0.163)	0.113 (0.101)	0.061 (0.153)			
Female	-0.101** (0.041)	-0.073 (0.076)	-0.064 (0.061)	-0.154** (0.076)	0.065 (0.040)		0.055 (0.074)	0.044 (0.059)	0.096 (0.071)			
Constant	-0.031 (0.062)	-0.076 (0.113)	-0.047 (0.086)	0.084 (0.134)	0.084 (0.065)		-0.034 (0.112)	-0.003 (0.100)	0.284** (0.129)			
Observations	5,753	1,909	1,912	1,932	5,753		1,909	1,912	1,932			
R-squared	0.006	0.002	0.015	0.013	0.012		0.008	0.014	0.040			

Note: ***significant at the 1% level; ** significant at the 5% level; *significant at the 10% level. SE clustered at the PSU level (census sector or segment) in parentheses. OLS Estimation. Controls include child’s sex and a set of child dummies for age categories in months (0-2, 3-5, 6-8, 9-11, 12-14, 15-17, 18-20, 21-23, 24-26, 27-29, 30-32, 33-35, 36-38, 39-41, 42-44, 45-47, 48-50, 51-53, 54-56, 57-59). Quintiles are population quintiles using monthly per capita household consumption as the ranking variable. *p*-value (*F*-test for quintile effects) is the *p*-value of the *F*-test of Q2=Q3=Q4=Q5=0.

**Table 11:** Adjusted SES gradients in child development by child’s age with IPW adjustments for missing data

	**Height for age z-score**				**Weight for age z-score**				**Weight for height z-score**			
VARIABLES	3-11 Months	12-23 Months	24-36 Months	37-59 Months	3-11 Months	12-23 Months	24-36 Months	37-59 Months	3-11 Months	12-23 Months	24-36 Months	37-59 Months
Quintile 2 = 1	0.130 (0.126)	0.290*** (0.091)	0.378*** (0.086)	0.316*** (0.068)	0.199* (0.104)	0.308*** (0.091)	0.269*** (0.078)	0.209*** (0.061)	0.158 (0.101)	0.196** (0.090)	0.102 (0.083)	0.027 (0.068)
Quintile 3 = 1	0.435*** (0.136)	0.601*** (0.091)	0.695*** (0.079)	0.758*** (0.069)	0.387*** (0.106)	0.533*** (0.087)	0.393*** (0.085)	0.511*** (0.066)	0.208* (0.112)	0.293*** (0.086)	0.020 (0.091)	0.079 (0.065)
Quintile 4 = 1	0.610*** (0.134)	0.827*** (0.108)	0.958*** (0.088)	0.774*** (0.072)	0.445*** (0.127)	0.566*** (0.100)	0.672*** (0.085)	0.538*** (0.061)	0.106 (0.128)	0.231** (0.101)	0.195** (0.088)	0.141** (0.063)
Quintile 5 = 1	0.616*** (0.145)	0.974*** (0.126)	1.139*** (0.099)	1.066*** (0.084)	0.513*** (0.131)	0.778*** (0.106)	0.743*** (0.085)	0.754*** (0.082)	0.223* (0.125)	0.391*** (0.107)	0.155* (0.085)	0.166** (0.076)
Female = 1	0.174** (0.082)	0.239*** (0.074)	0.160** (0.063)	-0.057 (0.052)	0.158** (0.073)	0.190*** (0.066)	0.116** (0.052)	-0.083 (0.051)	0.090 (0.073)	0.103* (0.061)	0.046 (0.053)	-0.054 (0.047)
Constant	-0.658*** (0.153)	-1.567*** (0.181)	-2.074*** (0.302)	-1.472*** (0.173)	-0.369*** (0.124)	-0.811*** (0.174)	-1.000*** (0.225)	-0.293 (0.204)	0.275** (0.128)	-0.196 (0.184)	0.012 (0.231)	0.584** (0.228)
Observations	1,718	2,391	2,411	3,882	1,693	2,285	2,325	3,707	1,689	2,285	2,323	3,702
R-squared	0.047	0.092	0.126	0.129	0.033	0.066	0.081	0.076	0.007	0.019	0.009	0.005
	**Stunting (<-2SD)**				**Underweight (<-2SD)**				**Overweight (>2SD)**			
VARIABLES	3-11 Months	12-23 Months	24-36 Months	37-59 Months	3-11 Months	12-23 Months	24-36 Months	37-59 Months	3-11 Months	12-23 Months	24-36 Months	37-59 Months
Quintile 2 = 1	-0.061* (0.032)	-0.150*** (0.038)	-0.118*** (0.039)	-0.118*** (0.026)	-0.010 (0.014)	-0.032*** (0.012)	-0.009* (0.005)	0.008 (0.011)	0.002 (0.022)	0.022 (0.017)	0.013 (0.019)	0.006 (0.014)
Quintile 3 = 1	-0.108*** (0.030)	-0.205*** (0.034)	-0.228*** (0.034)	-0.193*** (0.026)	-0.002 (0.018)	-0.033*** (0.012)	0.004 (0.009)	0.007 (0.007)	0.019 (0.027)	0.022 (0.017)	0.007 (0.017)	0.019 (0.017)
Quintile 4 = 1	-0.132*** (0.029)	-0.261*** (0.033)	-0.260*** (0.032)	-0.228*** (0.025)	-0.027** (0.012)	-0.031** (0.013)	-0.009* (0.005)	-0.008* (0.004)	0.013 (0.025)	0.006 (0.015)	0.036 (0.022)	0.025 (0.018)
Quintile 5 = 1	-0.098*** (0.034)	-0.285*** (0.031)	-0.298*** (0.033)	-0.255*** (0.023)	-0.029** (0.012)	-0.029** (0.014)	-0.006 (0.007)	-0.000 (0.006)	0.012 (0.030)	0.062*** (0.024)	0.040* (0.023)	0.070*** (0.020)
Female = 1	-0.047** (0.021)	-0.089*** (0.023)	-0.061*** (0.023)	0.025 (0.017)	-0.035*** (0.010)	-0.006 (0.007)	-0.004 (0.004)	-0.008 (0.006)	0.005 (0.019)	-0.002 (0.014)	-0.020 (0.017)	-0.025* (0.013)
Constant	0.216*** (0.030)	0.358*** (0.060)	0.566*** (0.090)	0.370*** (0.057)	0.069*** (0.016)	0.049** (0.023)	0.047** (0.021)	-0.010 (0.021)	0.109*** (0.027)	0.020 (0.034)	0.002 (0.064)	0.089* (0.052)
Observations	1,718	2,391	2,411	3,882	1,689	2,285	2,323	3,702	1,689	2,285	2,323	3,702
R-squared	0.031	0.073	0.072	0.059	0.022	0.010	0.005	0.005	0.003	0.010	0.006	0.011
	**Anemia**				**Vitamin A deficiency**		**Iron deficiency**					
VARIABLES	3-11 Months	12-23 Months	24-36 Months	37-59 Months	6-11 Months	12-23 Months	6-11 Months	12-23 Months				
Quintile 2 = 1	-0.039 (0.050)	-0.025 (0.036)	-0.097*** (0.034)	-0.096*** (0.033)	0.071 (0.095)	0.079 (0.059)	0.052 (0.078)	0.022 (0.055)				
Quintile 3 = 1	-0.103* (0.054)	-0.073* (0.038)	-0.245*** (0.051)	-0.165*** (0.040)	0.028 (0.080)	0.077 (0.060)	0.046 (0.070)	0.035 (0.050)				
Quintile 4 = 1	-0.137** (0.056)	-0.057 (0.041)	-0.228*** (0.043)	-0.234*** (0.039)	-0.056 (0.089)	-0.011 (0.069)	0.052 (0.071)	0.044 (0.066)				
Quintile 5 = 1	-0.310*** (0.052)	-0.254*** (0.044)	-0.284*** (0.042)	-0.285*** (0.038)	-0.119 (0.078)	-0.061 (0.054)	-0.094* (0.055)	-0.054 (0.048)				
Female = 1	-0.074** (0.033)	-0.038 (0.026)	-0.000 (0.030)	-0.082*** (0.024)	-0.012 (0.048)	-0.003 (0.038)	-0.120** (0.047)	-0.099*** (0.036)				
Constant	0.440*** (0.063)	0.921*** (0.071)	1.205*** (0.126)	1.102*** (0.086)	0.381** (0.151)	0.216** (0.106)	0.080 (0.109)	0.543*** (0.086)				
Observations	1,488	2,167	2,179	3,580	596	994	596	994				
R-squared	0.089	0.040	0.057	0.057	0.020	0.016	0.048	0.037				
	**Gross motor z-score**			**Communication z-score**								
VARIABLES	3-11 Months	12-23 Months	24-36 Months	3-11 Months	12-23 Months	24-36 Months						
Quintile 2 = 1	-0.035 (0.101)	-0.068 (0.099)	0.183 (0.111)	-0.157 (0.108)	-0.290*** (0.089)	0.068 (0.092)						
Quintile 3 = 1	-0.029 (0.107)	-0.036 (0.085)	0.127 (0.120)	0.200** (0.097)	-0.259*** (0.085)	0.225** (0.089)						
Quintile 4 = 1	-0.040 (0.102)	0.012 (0.118)	0.130 (0.113)	-0.014 (0.107)	-0.215** (0.103)	-0.032 (0.111)						
Quintile 5 = 1	-0.013 (0.111)	0.144 (0.095)	0.404*** (0.116)	0.200 (0.123)	-0.114 (0.085)	0.228* (0.125)						
Female = 1	-0.135** (0.060)	-0.048 (0.066)	-0.094 (0.070)	0.004 (0.064)	0.141** (0.063)	0.076 (0.059)						
Constant	-0.149 (0.112)	-0.164 (0.154)	-0.147 (0.248)	0.078 (0.120)	0.153 (0.158)	-0.471 (0.290)						
Observations	1,476	2,185	2,092	1,476	2,185	2,092						
R-squared	0.011	0.007	0.019	0.021	0.017	0.014						

Note: ***significant at the 1% level; ** significant at the 5% level; *significant at the 10% level. SE clustered at the PSU level (census sector or segment) in parentheses. OLS Estimation. Controls include child’s sex and a set of child dummies for age categories in months (0-2, 3-5, 6-8, 9-11, 12-14, 15-17, 18-20, 21-23, 24-26, 27-29, 30-32, 33-35, 36-38, 39-41, 42-44, 45-47, 48-50, 51-53, 54-56, 57-59). Quintiles are population quintiles using monthly per capita household consumption as the ranking variable. *p*-value (*F*-test for quintile effects) is the *p*-value of the *F*-test of Q2=Q3=Q4=Q5=0.

## Appendix 2

### Technical Appendix: Construction of SES indicators

#### Direct measure of SES: household consumption

Household consumption was used as our preferred direct measure of SES. In our context, we believe consumption is a better measure of SES than other monetary measures such as income, considering the problems with measuring income in settings with a high proportion of self-employed and informal workers. In addition, irregular and intermittent earnings from informal employment make income measurements more volatile than consumption and, therefore, more directly related to current living standards [22, 35].

Our consumption index is based on the aggregation of payments for goods and services collected using monthly, quarterly, and yearly reference periods. For food consumption, we used information about monthly purchases of a list of 41 food items as well as spending on food consumed outside the house. Because home-produced foods are an important part of food consumption in rural areas, we included self-reported valuations of consumption from household production. Nonfood consumption includes payments in housing, household services, education, personal goods, health, transport, recreation, and financial services. For non-renters, housing consumption was imputed using a hedonic price model. We excluded spending on durable goods and other lumpy spending such as hospitalizations^18^. To construct total consumption, individual items based on quarterly and yearly recall periods were converted into monthly figures and then all food and nonfood items were added together. We adjusted for household size by dividing total consumption by the number of household members and obtaining a per capita measure.

#### Proxy measure of SES: wealth index

Additionally, we developed an alternative proxy measure of SES by estimating a composite indicator “wealth index” that combines information about ownership of household assets, physical characteristics of the dwelling and access to basic services using principal components analysis [26, 36]^19^. Asset-based indices have several advantages over direct measures of living standards: data on assets and dwelling characteristics are less expensive and easier to collect; they are more robust to measurement error and reporting biases than income and expenditures; and an asset-based index reflects the notion of permanent income more closely, and is based on a long term conceptualization of wealth, which is more relevant for inequality analysis ([36, 37]; O’Donnell et al., 2008 [26, 38];). Wealth indices based on principal components analysis have also been used in analyses of socioeconomic gaps in health and child development outcomes in low- and middle-income countries [3, 4, 6, 26, 39]. For this study, a wealth index was estimated based on assets ownership, including refrigerator, radio, television, fixed phone line, car, motorcycle, bicycle; and information about water sources, toilet facilities, electricity, and the construction material of wall, roof, and floor in the household^20^. The resulting score is a standardized wealth index with a mean of zero and a standard deviation of one. All members in the household receive the same wealth index score. Figure 2 shows the distribution of households by the value of the wealth index.

## Footnotes

1 According to Grantham-McGregor et al. [2], the degree of loss of developmental potential is the discrepancy between children’s developmental levels and what they would have achieved in a more nurturing environment with adequate stimulation and nutrition.

2 Such as involvement in violent behavior (physical fight, gun use, gang membership).

3 Following the framework described in Walker et al. [11], these factors can be grouped in biological risks (such as undernutrition, micronutrient deficiencies and disease) and psychosocial risks (parenting and contextual factors) that affect children’s development through changes in brain function and behavior.

4 The WHO Global Health Observatory country estimate for 2016 is 46.9% [23.3 – 70.0], the second highest rate in the region, only after Haiti and more than double the regional average of 22.7% (https://apps.who.int/gho/data/node.main.ANEMIACHILDREN?lang=en).

5 The survey was conducted to evaluate the effect of two national social programs: the Bono Juana Azurduy and the Zero Malnutrition Program.

6 In a first stage, 424 clusters were selected (300 rural and 124 urban); in a second stage, 20 eligible households were randomly selected within each cluster.

7 Z-score = (observed value - median value of the reference population) / standard deviation value of reference population.

8 The WHO reference growth standards were established in 2006 and represent the growth patterns of healthy children from different regions of the world.

9 For children between 6 and 59 months of age we used standard WHO cutoff values: mild anemia, Hb 10.0 - 10.9 g/dL; moderate anemia, Hb 7.0 - 9.9 g/dL; severe anemia, Hb <7.0 g/dL. Because there is no standard cutoff to define anemia among infants 3 to 5 months, we used a cutoff of 10.5 g/dl following Chandyo et.al. (2016).

10 DBS were collected at the end of the interview after caregiver’s consent. Samples were identified and stored in hermetic plastic containers with drying agents to accelerate the dehydration process and to control humidity during transportation. Once dry they were packed using Ziploc bags. The DBS method had several advantages in field work. First, its simplicity and micro volume of blood required makes it appropriate for its use in small children, with very low health and biological risks. Second, the technique does not require specialized health personnel, intensive training or equipment. Third, DBS are stable to normal outside temperature for relatively long periods, which allows sufficient time to transport samples from rural areas and then to laboratories. In addition, it does not require special transportation conditions (such as cold chain), except humidity control and excess heath, which can both damage samples. Blood samples for DBS were collected separately from samples to determine hemoglobin concentrations.

11 For analysis, all samples were flown to a specialized laboratory at the University of Giessen, Germany.

12 From the subsample of 2,000 children 6-23 months selected for the DBS study, the ESNUT was able to collect 1,701 valid blood samples (the rest had either quality problems or were not available for analysis). In addition, 46 samples had to be discarded due to identification issues (incorrect household or child id).

13 Age groups were: 3.6-6.5 months; 6.6 to 9.5 months; 9.6-11.5 months; 11.6-13.5 months; 13.6-15.5 months; 15.6-17.5 months; 17.6-19.5 months; 19.6-22.5 months; 22.6-24.5 months; 24.6-30.5 months; 30.6-36.5 months.

14 The ASQ-2 is a developmental screening tool for children between 4 and 60 months, comprising 19 age-specific questionnaires designed to screen infants and young children for developmental delays during the first 5 years of life (Ages & Stages Questionnaires, Second Edition. A Parent-Completed, Child Monitoring System, by Diane Bricker and Jane Squires. Copyright © 1999 by Paul H. Brookes Publishing Co.).

15 Following Rutstein and Johnson [27], the quintile cutoffs are based on total household population, instead of the households themselves. Quintiles were constructed using a weighted frequency distribution of households where weights were the product of the number of de jure household members and the sampling weight of the household. Consequently, a tabulation of the unweighted sample will not produce five quintile groups of equal size, but the weighted sample will.

16 We test whether the coefficients for the quintile indicators are jointly zero.

17 Note, however, that Rubio-Codina et al (2016) internal validity assessment is based on the ASQ-3.

## References

[1] Lu C, Black MM, Richter LM (2016). Risk of poor development in young children in low-income and middle-income countries: an estimation and analysis at the global, regional, and country level. Lancet Glob Health.

[2] Grantham-McGregor S, Cheung YB, Cueto S, Glewwe P, Richter L, Strupp B (2007). Developmental potential in the first 5 years for children in developing countries. Lancet..

[3] Hamadani JD, Tofail F, Huda SN, Alam DS, Ridout DA, Attanasio O, Grantham-McGregor SM. Cognitive Deficit and Poverty in the First 5 Years of Childhood in Bangladesh. Pediatrics. 2014;134(4). 10.1542/peds.2014-0694 25266433

[4] Rubio-Codina M, Attanasio O, Meghir C, Varela N, Grantham-McGregor S (2015). The socio-economic gradient of child development children 6-42 months in Bogota the socio-economic gradient of. J Human Res.

[5] Schady N, Behrman JR, Araujo MC, Azuero R, Bernal R, Bravo D (2015). Wealth Gradients in Early Childhood Cognitive Development in Five Latin American Countries. J Human Res.

[6] Van De Poel E, Hosseinpoor AR, Speybroeck N, Van Ourti T, Vega J (2008). Socioeconomic inequality in malnutrition in developing countries. Bull World Health Organ.

[7] Naudeau S, Kataoka N, Valerio A, Neuman MJ, Elder LK (2011). Investing in young children: an early childhood development guide for policy dialogue and project preparation.

[8] Walker SP, Chang SM, Vera-Hernandez M, Grantham-McGregor S (2011). Early Childhood Stimulation Benefits Adult Competence and Reduces Violent Behavior. Pediatrics..

[9] Walker SP, Wachs TD, Grantham-Mcgregor S, Black MM, Nelson CA, Huffman SL (2011). Inequality in early childhood: Risk and protective factors for early child development. Lancet..

[10] Almond D, Currie J (2011). Chapter 15 – Human capital development before age five. Handbook of Labor Economics.

[11] Walker SP, Wachs TD, Meeks Gardner J, Lozoff B, Wasserman GA, Pollitt E, Carter JA (2007). Child development: risk factors for adverse outcomes in developing countries. Lancet.

[12] Black RE, Victora CG, Walker SP, Bhutta ZA, Christian P, De Onis M (2013). Maternal and child undernutrition and overweight in low-income and middle-income countries. Lancet..

[13] Lozoff B, Beard J, Connor J, Felt B, Georgieff M, Schallert T (2006). Long-Lasting Neural and Behavioral Effects of Iron Deficiency in Infancy. Nutr Rev.

[14] West KP, Darnton-Hill I, Semba RD, Bloem MW (2001). Vitamin A Deficiency. Nutrition and Health in Developing Countries.

[15] INE-Ministerio de Salud (2017). Encuesta de Demografía y Salud EDSA 2016. La Paz.

[16] Grantham-McGregor S, Ani C (2001). A Review of Studies on the Effect of Iron Deficiency on Cognitive Development in Children. J Nutr..

[17] World Health Organization (2006). WHO child growth standards: lenght/height-for-age, weight-for-age, weight-for-length, weight-for-height and body mass index-for-age: methods and development.

[18] Erhardt JG, Estes JE, Pfeiffer CM, Biesalski HK, Craft NE (2004). Combined measurement of ferritin, soluble transferrin receptor, retinol binding protein, and C-reactive protein by an inexpensive, sensitive, and simple sandwich enzyme-linked immunosorbent assay technique. J Nutr.

[19] Sommer A, Davidson FR (2002). Assessment and Control of Vitamin A Deficiency: The Annecy Accords. J Nutr.

[20] Fernald LCH, Kariger P, Hidrobo M, Gertler PJ (2012). Socioeconomic gradients in child development in very young children: Evidence from India, Indonesia, Peru, and Senegal. Proc Natl Acad Sci.

[21] Rubio-Codina M, Araujo MC, Attanasio O, Muñoz P, Grantham-McGregor S (2016). Concurrent validity and feasibility of short tests currently used to measure early childhood development in large scale studies. PLoS ONE.

[22] Rubio-Codina M, Attanasio O, Grantham-McGregor S (2016). Mediating pathways in the socio-economic gradient of child development. Int J Behav Dev.

[23] O’Donnell O, Van Doorslaer E, Wagstaff A, Lindelow M (2008). Analyzing health equity using household survey data: a guide to techniques and their implementation.

[24] Wagstaff A, Watanabe N (2003). What difference does the choice of SES make in health inequality measurement?. Health Econ.

[25] Lindelow M (2006). Sometimes more equal than others: How health inequalities depend on the choice of welfare indicators. Health Econ.

[26] Houweling TAJ, Kunst AE, Mackenbach JP (2003). Measuring health inequality among children in developing countries: Does the choice of the indicator of economic status matter?. Int J Equity Health.

[27] Rutstein SO, Johnson K (2004). The DHS Wealth Index. DHS Comparative Reports No. 6.

[28] World Health Organization (2013). Handbook on health inequality monitoring: with a special focus on low- and middle-income countries.

[29] Wooldridge JM (2007). Inverse probability weighted estimation for general missing data problems. J Econ.

[30] Campbell F, Conti G, Heckman JJ, Moon SH, Pinto R, Pungello E, Pan Y (2014). Early Childhood Investments Substantially Boost Adult Health. Science..

[31] Gertler P, Heckman J, Pinto R, Zanolini A, Vermeersch C, Walker S (2014). Labor market returns to an early childhood stimulation intervention in Jamaica. Science..

[32] Krawinkel MB, Assad-García J (2012). Estimación de los niveles de hierro y vitamina A en niños de 6 a 23 meses de edad en Bolivia. Reporte de la encuesta nutricional realizada de mayo a diciembre 2012 (unpublished report).

[33] Yip R, Semba R, Bloem MW (2001). Iron Deficiency and Anemia. Nutrition and Health in Developing Countries.

[34] Johannsen J, Martinez S, Vidal C, Yarygina A. Evaluación de impacto del programa de desarrollo infantil temprano “Crecer Bien para Vivir Bien”: modalidad visitas domiciliarias (Technical Notes No. IDB-TN-1790). Washington, D.C; 2019.

[35] Deaton A, Grosh M, Grosh M, Glewwe P (2000). Consumption. Designing Household Survey Questionnaires for Developing Countries: Lessons from Ten Years of LSMS Experience.

[36] Filmer D, Pritchet L (2001). Estimating Wealth Effects Without Expenditure Data—Or Tears: An Application to Educational Enrollments in States of India. Demography..

[37] Gakidou E, Oza S, Vidal Fuertes C, Li AY, Lee DK, Sousa A, et al. Improving Child Survival Through Environmental and Nutritional Interventions. The importance of Targeting Interventions Toward the Poor. Jama. 2007;298(16). 10.1001/jama.298.16.1876 17954539

[38] Vyas S, Kumaranayake L (2006). Constructing socio-economic status indices: How to use principal components analysis. Health Policy Plan.

[39] Gwatkin DR, Rutstein S, Johnson K, Suliman E, Wagstaff A, Amouzou A (2007). Socio-economic differences in health, nutrition, and population within developing countries. An overview. Country reports on HNP and poverty.

